# Genome Filtering for New DNA Biomarkers of *Loa loa* Infection Suitable for Loop-Mediated Isothermal Amplification

**DOI:** 10.1371/journal.pone.0139286

**Published:** 2015-09-28

**Authors:** Catherine B. Poole, Laurence Ettwiller, Nathan A. Tanner, Thomas C. Evans, Samuel Wanji, Clotilde K. S. Carlow

**Affiliations:** 1 New England Biolabs, Ipswich, Massachusetts, United States of America; 2 Research Foundation in Tropical Diseases and Environment, Buea, Cameroon; George Washington University School of Medicine and Health Sciences, UNITED STATES

## Abstract

*Loa loa* infections have emerged as a serious public health problem in patients co-infected with *Onchocerca volvulus* or *Wuchereria bancrofti* because of severe adverse neurological reactions after treatment with ivermectin. Accurate diagnostic tests are needed for careful mapping in regions where mass drug administration is underway. Loop-mediated isothermal amplification (LAMP) has become a widely adopted screening method because of its operational simplicity, rapidity and versatility of visual detection readout options. Here, we present a multi-step bioinformatic pipeline to generate diagnostic candidates suitable for LAMP and experimentally validate this approach using one of the identified candidates to develop a species-specific LAMP assay for *L*. *loa*. The pipeline identified ~140 new *L*. *loa* specific DNA repeat families as putative biomarkers of infection. The consensus sequence of one family, repeat family 4 (RF4), was compiled from ~ 350 sequences dispersed throughout the *L*. *loa* genome and maps to a *L*. *loa-*specific region of the long terminal repeats found at the boundaries of *Bel/Pao* retrotransposons. PCR and LAMP primer sets targeting RF4 specifically amplified *L*. *loa* but not *W*. *bancrofti*, *O*. *volvulus*, *Brugia malayi*, human or mosquito DNA. RF4 LAMP detects the DNA equivalent of one microfilaria (100 pg) in 25–30 minutes and as little as 0.060 pg of *L*. *loa* DNA (~1/1600^th^ of a microfilaria) purified from spiked blood samples in approximately 50 minutes. In summary, we have successfully employed a bioinformatic approach to mine the *L*. *loa* genome for species-specific repeat families that can serve as new DNA biomarkers for LAMP. The RF4 LAMP assay shows promise as a field tool for the implementation and management of mass drug administration programs and warrants further testing on clinical samples as the next stage in development towards this goal.

## Introduction

*Loa loa*, the African eye worm, is a filarial nematode parasite responsible for the neglected tropical disease loiasis. The parasite is transmitted to humans by tabanid *Chrysops* flies (primarily *C*. *silacea* and *C*. *dimidiate*) [1]. Between three and 13 million people are infected [2] in forested and some savannah regions of Western and Central Africa [3] with approximately 30 million at risk [4, 5]. Historically, loiasis has received less attention than other filarial diseases since it is generally asymptomatic. In some individuals, the migration of adult worms (30–70 mm in length) through the subconjunctiva of the eye; transient, localized angioedema (Calabar swelling); and pruritus are observed, with more serious clinical manifestations occasionally evident with chronic infection [1]. Sheathed microfilariae (mf) predominantly migrate between the lungs and peripheral blood but have also been recovered from spinal fluid, urine and sputum [1]. While approximately 40% of the infections are amicrofilaraemic [6, 7], certain infected individuals harbor more than 30,000 mf per ml of blood [8–12]. The disease has emerged as a serious public health problem because of severe adverse effects (SAEs) when mf titers are as low as 8000/ml [9], and even death in individuals with high mf levels (> 50,000 mf/ml) after treatment with ivermectin for onchocericasis [1, 12] or lymphatic filariasis [3, 13–15]. The risk of co-infection necessitates careful mapping of *L*. *loa*, *O*. *volvulus* and *W*. *bancrofti* infections [3, 13–16]. *L*. *loa* infected individuals are treated with diethylcarbamazine, which is active against adults and mf [17] followed by albendazole to eliminate residual mf [18].

A history of eye worm and/or the occurrence of Calabar swelling are used as an indication of infection, but detection of mf is required for definitive diagnosis [1]. Parasitological diagnosis based on microscopy [19] is challenging as mf can only be detected in the peripheral blood between the daytime hours of 10:00–16:00 and many infections are occult [6, 7]. Differentiation from other species of mf is based on the presence of a sheath, and the staining pattern of nuclei in the tail. While microscopy is a valuable technique, morphological interpretation can be subjective and requires substantial expertise. More recently, hand-held mf detection devices, which score motility, have been described [11, 20]. However, these methods are not useful for occult infections, nor will they distinguish *L*. *loa* mf from other blood-borne mf.

Serological assays measuring *L*. *loa* specific antibody responses to the recombinant antigens *Ll-*SXP-1 [21] and repeat 3 of the *L*. *loa* nematode polyprotein antigen [22] have been proposed for the detection of occult infections [22, 23].

Several DNA targets have been described for use in PCR, including repeat 3 of the nematode polyprotein antigen [24–26] LLMF72 [27]; the LL3M9 repeat family [27, 28]; and the internal transcribed spacer 1 region of rDNA [29]. These targets are either present in low copy number, which can impact sensitivity, or are not species-specific.

Several isothermal amplification methods targeting DNA have been developed which offer significant advantages over PCR. Of these methods, loop-mediated isothermal amplification (LAMP) has become the most widely adopted. Its simplicity and visual detection format without the need for expensive equipment offers considerable advantages over PCR [30–32]. Two recent publications have described LAMP assays for *L*. *loa* using the PCR targets, LL3M9 and LLMF72 [33, 34]. However, these are not necessarily the ideal targets for this platform. LL3M9 contains multiple copies of a simple 6 bp repeat which is conserved in nematodes, and LLMF72 is a single copy gene. These attributes may affect specificity and sensitivity respectively.

The genomic era has brought with it the ability to devise comparative and subtractive strategies to identify, *in silico*, new diagnostic candidates from an organism’s complete genome. In the present study, we devise a multi-step bioinformatic pipeline to identify species-specific DNA repeat sequences that are particularly suited for LAMP and experimentally validate the approach using one of the biomarkers to develop a new diagnostic test for *L*. *loa*.

## Materials and Methods

### Materials

Genomic DNA samples were generously donated by the following: *L*. *Loa*, B.L. Makepeace and C. Hartley (University of Liverpool); *B*. *malayi*, L.A. McReynolds (New England Biolabs); *O*. *volvulus* and *Homo sapiens*, F. Perler (New England Biolabs); *Aedes albopictus*, Z. Li (New England Biolabs). Whole genome amplified (WGA) *W*. *bancrofti* DNA was obtained from the NIH/NIAID Filariasis Research Reagent Resource Center (http://www.filariasiscenter.org). Human whole blood was obtained from Innovative Research (Novi, MI). DNA quantity was determined using a Qubit dsDNA HS Assay kit in conjunction with a Qubit 2.0 Fluorometer as directed by the manufacturer (Life Technologies).

Due to limited quantities of genomic *L*. *loa* DNA, WGA *L*. *loa* DNA was generated using the PicoPLEX WGA Kit as instructed by the manufacturer (Rubicon Genomics) and used as the primary DNA source for *L*. *loa* LAMP assay development.

### Bioinformatic Analysis

**Fig 1:**
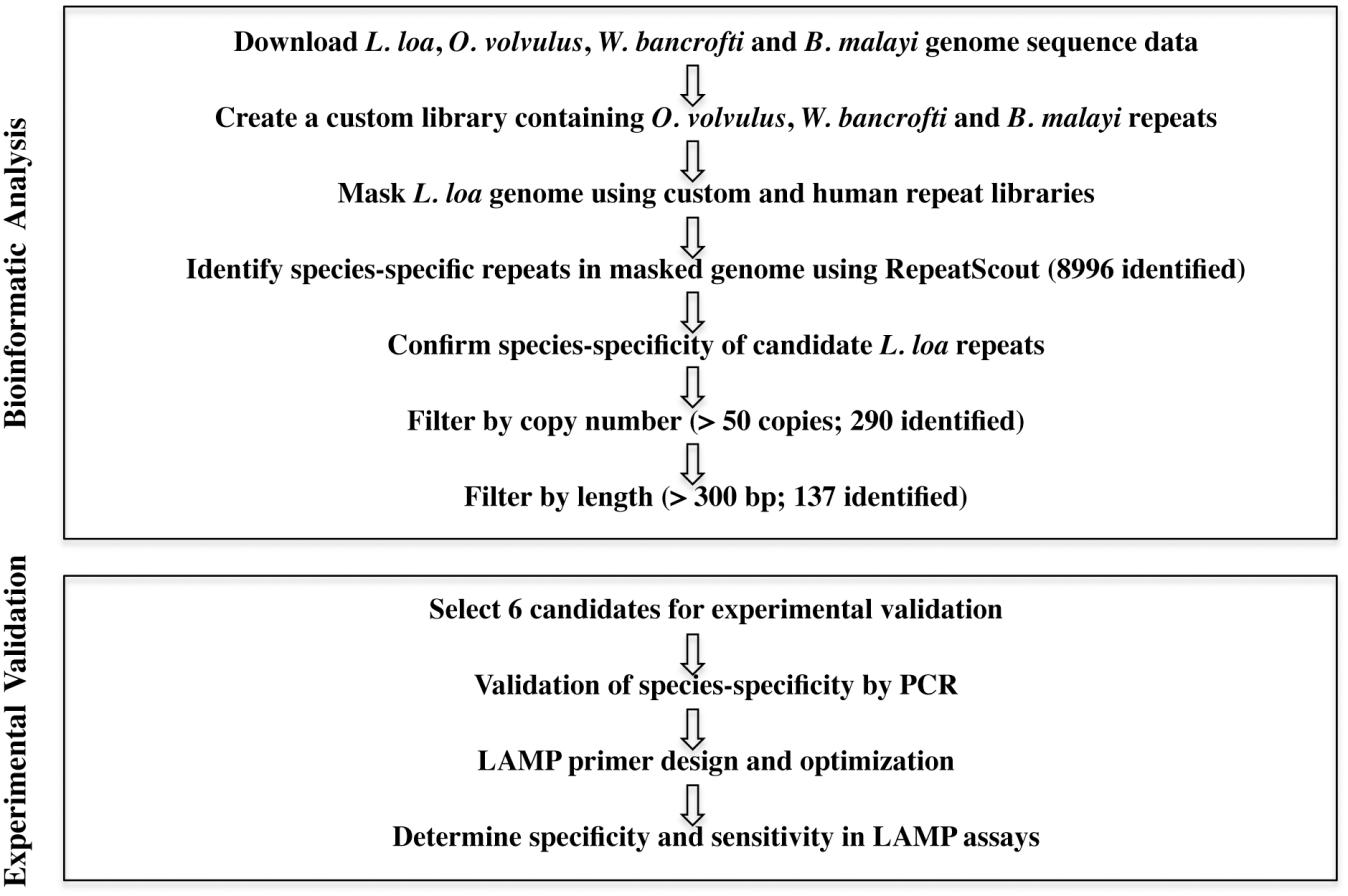
Bioinformatic filtering pipeline to identify *L*. *loa*-specific repeat families. Genome sequences were downloaded from the Filarial Worms sequencing project, Broad Institute of Harvard and MIT (http://www.broadinstitute.org/). The *L*. *loa* genome was masked using RepeatMasker then mined for *L*. *loa*-specific repeats using RepeatScout. The resulting consensus repeat sequences that consist of 51 or more members and that are 300 bp or more in length were selected for further evaluation.

Genome sequence data for *L*. *loa* (V3), *B*. *malayi*, *W*. *bancrofti* and *O*. *volvulus* were downloaded from the Filarial worms Sequencing Project, Broad Institute of Harvard and MIT (http://www.broadinstitute.org/). For each genome, contig and supercontigs were assembled into a single file. RepeatScout (version 1.0.5) [35] was run on the *O*. *volvulus*, *B*. *malayi* and *W*. *bancrofti* genomes to identify repeat sequences using the following parameters: build _lmer_table-l 15 followed by RepeatScout using the default parameters, and the 15 mer table obtained using the build_lmer_table algorithm. Next, the repeats identified in *B*. *malayi*, *W*. *bancrofti* and *O*. *volvulus* using RepeatScout were combined into a single custom library. Then, RepeatMasker was run on the *L*. *loa* genome using the custom-repeat library followed by a human repeat library (http://repeatmasker.org) [36, 37]. RepeatScout was then run on the masked *L*. *loa* genome described above generating a library containing putatively specific *L*. *loa* consensus repeat sequences. To confirm their specificity, candidates were screened against the filarial genomes using the following blastn parameters: outfmt = 5, num_ alignments = ´10000000´, word_size = 15, *e*-values less than 10e-7. Only sequences absent from the *O*. *volvulus*, *B*. *malayi* and *W*. *bancrofti* genomes but represented by at least 51 copies in the *L*. *loa* genome were further processed. To facilitate LAMP primer design, only consensus sequences longer than 300 bp were retained and sorted by percent GC content (Fig 1).

Contigs (GenBank accession numbers: JPEI01001237.1, JPEI01001554.1, JPEI01001218.1, JPEI01001588.1, JPEI01001706.1) containing multiple repeat family 4 (RF4) members were aligned with themselves using Blastn (http://blast.ncbi.nlm.nih.gov/) or with other RF4 containing contigs (if only one family member was present) to confirm repeat length. Blastx analysis (http://blast.ncbi.nlm.nih.gov/) as well as the Gypsy Database 2.0 (www.gydb.org) and Censor at Repbase (www.girinst.org/censor/index.php, [38]) were used to characterize and map the conserved proteins identified adjacent to RF4 members in the *L*. *loa* genome.

### Primer Design

**Fig 2:**
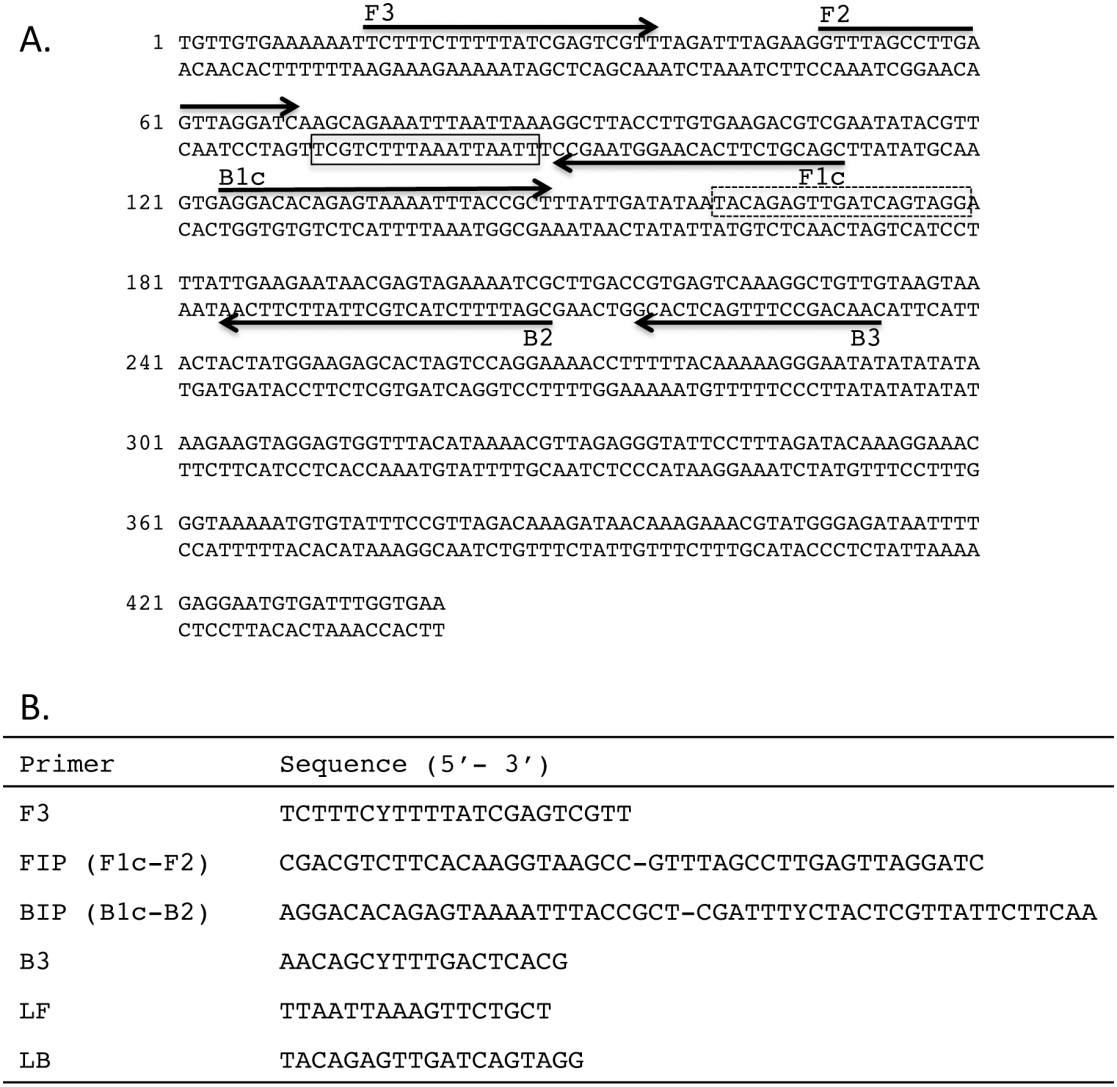
*L*. *loa* LAMP primer set targeting RF4. Location of the six LAMP primers within the 440 bp consensus sequence of RF4 is shown **(A)**. Arrows indicate the direction of extension. Solid and dash line boxes represent the binding regions of the loop forward (LF) and loop back (LB) primers respectively. **(B)** Sequence of the RF4 LAMP primers. F3, BIP and B3 are degenerate oligonucleotides where Y = C or T.

PCR primers were designed to amplify repeat families using the consensus sequences generated by RepeatScout. The forward and reverse degenerate primer sequences for *L*. *loa* RF4 are (5’ TCTTTCYTTTTATCGAGTCGTT 3’) and (5’ TCYTYAAAATTATCTCCCATACG 3’) respectively, where Y = C or T. For LAMP primer design, the *L*. *loa* RF4 consensus sequence was submitted to PrimerExplorer V4 (http://primerexplorer.jp/e/) generating primer sequences for F3, FIP, BIP and B3 (Fig 2). Loop primers (Fig 2) were designed manually using ‘‘A guide to LAMP primer design” available from the Eiken Chemical Co. (http://primerexplorer.jp/e/). Primers were synthesized (PCR and LAMP) and HPLC purified (LAMP) by Integrated DNA Technologies (Coralville, IA, USA).

### PCR Assays

DNA (20 ng) was mixed with 12.5 μl of One Taq Hot Start 2X Master Mix with standard buffer (New England Biolabs) containing 0.2 mM each of the forward and reverse *L*. *loa* RF4 primers in a final volume of 25 μl. Reactions were denatured once at 94°C for 30 sec then cycled 30 times at 94°C for 30 sec, 51°C for 30 sec and 68°C for 30 sec followed by a final 5 min extension at 68°C.

As a positive control for the presence of intact DNA, a 244 bp actin fragment was PCR amplified from 1 ng of various DNAs using One Taq Hot Start 2X Master Mix with standard buffer (New England Biolabs) in 25 μl reactions containing 3.6 mM MgCl_2_ and 0.2 μM each of the forward and reverse actin primers [39]. When mosquito or human DNA was used as template, reactions contained 9 ng of DNA and 4.4 mM MgCl_2_. All reactions were denatured once at 95°C for 30 sec then cycled 35 times at 95°C for 30 sec, 50°C for 30 sec and 68°C for 30 sec followed by a final 5 min extension at 68°C.

Reaction products were analyzed by electrophoresis on 1.5% agarose gels equilibrated with 1X Tris-borate/EDTA buffer [40].

### LAMP Assays

LAMP reactions contained 1.6 μM each of primers FIP and BIP, 0.2 μM each of F3 and B3, 0.4 μM each of LF and FB, 1.4 mM of each dNTP, 20 mM Tris-HCl (pH 8.8), 50 mM KCl, 10 mM (NH_4_)_2_SO_4_, 8 mM MgSO_4_, 0.1% Tween-20 and 8 U of *Bst* 2.0 DNA Polymerase (New England Biolabs) mixed with one of several template DNAs, or 10 mM Tris-HCl (pH 8), 0.1 mM EDTA for non-template controls (NTC), in a total volume of 25 μl. One μl of a 25X Valeramide (V) N,N-Diethylformamide (DEF) solution (final concentration of 34mM V/128 mM DEF) was added to some reactions to reduce non-specific amplification [41, 42]. Reactions were incubated at 61°C for 60–90 minutes in a Loopamp Realtime Turbidimeter (LA-320c, Eiken Chemical Co.). The instrument measures the change in turbidity at 650 nm caused by the precipitation of magnesium pyrophosphate produced by polymerase activity. Turbidity data were analyzed using the LA-320c software package that reports when the change in turbidity over time (dT/dt) reaches a value of 0.1, which we then assigned to be the threshold time (Tt).

To mimic a clinical situation, a series of blood samples spiked with *L*. *loa* DNA were prepared for analysis with LAMP. Four ng of genomic *L*. *loa* DNA were diluted to 20 μl with uninfected human whole blood then a set of two-fold serial dilutions was prepared from this spiked sample using additional blood. For the non-template control, a sample containing only uninfected human whole blood was prepared. DNA was extracted from each 10 μl dilution using the ChargeSwitch gDNA Blood Kit (Invitrogen) as directed by the manufacturer and the purified DNA was eluted in 25 μl of 10 mM Tris-HCl, pH 8.5. Two μl of each dilution was used in LAMP reactions containing the V/DEF chemical additive. The quantity of *L*. *loa* DNA amplified from spiked blood samples was determined from a standard curve generated by amplifying known amounts of genomic *L*. *loa* DNA using the same LAMP conditions.

## Results

### Identification and Evaluation of Diagnostic Candidates

The assembled *L*. *loa* genome [43] enabled an *in silico* search for new DNA biomarkers. A bioinformatic pipeline was constructed to identify species-specific *L*. *loa* repeats (Fig 1). The first step towards this objective was to mask the *L*. *loa* genome for repeats common to filarial parasites. Approximately 12% (11.92%) of the *L*. *loa* genome was masked using the custom filarial repeat library created from *B*. *malayi*, *W*. *bancrofti* and *O*. *volvulus* sequence data with RepeatScout. An additional 4% (3.74%) of the *L*. *loa* genome was masked using the human repeat library as well as with simple repeats identified by RepeatMasker. Following masking of the *L*. *loa* genome, a total of 8996 putatively specific *L*. *loa* consensus repeat sequences were identified using RepeatScout. To confirm species-specificity, diagnostic candidates were screened against the filarial genomes using Blastn. As copy number may influence assay sensitivity [44, 45], the data was filtered for those families consisting of 51 or more copies. The 290 families identified were then filtered by size (>300 bps) to ease LAMP primer design. A total of 137 *L*. *loa* repeat families were identified that fulfilled our criteria, representing potential new DNA biomarkers suitable for a LAMP based assay (S1 Text).

Six *L*. *loa* repeat families (RF0, RF4, RF39, RF683, RF972 and RF1628) were chosen for further evaluation based on copy number (368–774) to maximize sensitivity, as well as % GC content (33–35) to facilitate primer design (S1 Text). Multiple primer sets were designed to the RepeatScout consensus sequence generated for each repeat family and tested empirically in both PCR and LAMP amplification assays. All except RF4 were eventually eliminated from consideration (data not shown) due to either poor amplification (RF1628), complex banding patterns in PCR (RF0, RF39), or poor sensitivity in LAMP reactions (RF683, RF972).

**Fig 3:**
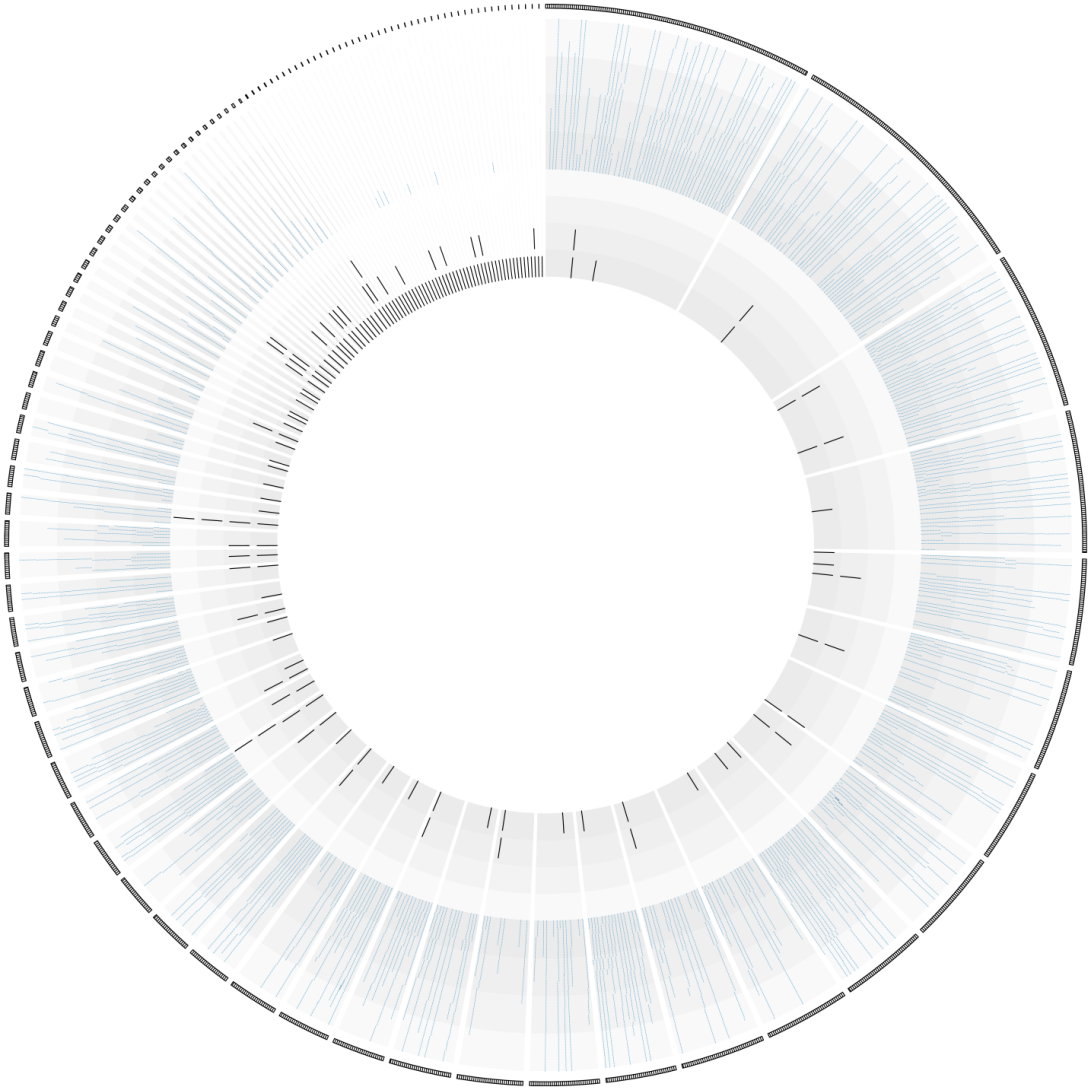
Spatial distribution of RF4 members in the *L*. *loa* genome. Circos representation of all the contigs from the *L*. *loa* genome (sorted by size around the outer edge) where copies of RF4 are located (black spokes around inner circle) and their position within each contig.

RepeatScout identified 368 members of RF4 in the *L*. *loa* genome [43] from which it compiled a 440 bp long, 33% GC rich consensus sequence (S1 Fig). Blastn analysis of a more recently released version of the *L*. *loa* genome generated using Single Molecule, Real-Time DNA sequencing [46] with the RF4 consensus sequence identified 350 members of this repeat. Spatial distribution of RF4 members in each of the contigs from the *L*. *loa* genome revealed that most occur as singletons. The remainder is found adjacent to each other, though not arranged in tandem arrays (Fig 3). Many of the larger contigs contain multiple copies of two (34 occurrences), three (five occurrences) or a maximum of four repeats (two occurrences).

RF4 comprises part of the Long Terminal Repeats (LTRs) in *Bel/Pao* retrotransposons [47]. Examination of the genomic sequence surrounding RF4 members demonstrated that they span part of a larger (~ 600 bp) repetitive sequence within the *L*. *loa* genome (S2 Fig). Blastx analysis of the sequence in the vicinity of these 600 bp repetitive regions revealed conserved proteins exhibiting high identity with the Mabel family of *Bel/Pao* retrotransposons in *B*. *malayi* (Gypsy Database 2.0: www.gydb.org; Censor at Repbase: (www.girinst.org/censor/index.php, [38]) suggesting that RF4 comprises part of the LTR at the ends of Bel/Pao retrotransposons in *L*. *loa*. A detailed examination of one *L*. *loa* scaffold, (GenBank acc. # JPEI01001237.1) revealed two 600 bp LTRs bordering the sequence coding for the putative proteins of this 7.9 kb retrotransposon (S2 Fig). Alignment of the LTR and RF4 consensus sequences (data not shown) demonstrated that two non-contiguous regions of the LTR (nts 75–150 and 230–600) are species-specific and appear to have been fused by RepeatScout to generate the RF4 consensus sequence (S2 Fig). Blastn analysis of the *L*. *loa* LTR consensus sequence demonstrated that the regions of the LTR extending from nts 1 to ~70 and from ~170–290 exhibit homology with the *B*. *malayi*, *O*. *volvulus* and *W*. *bancrofti* genomes and thus were excluded from the RF4 consensus. The LAMP primers map between nts 360–600, well within the species-specific region of the *L*. *loa* LTR.

### Specificity of RF4 in DNA Amplification Assays

**Fig 4:**
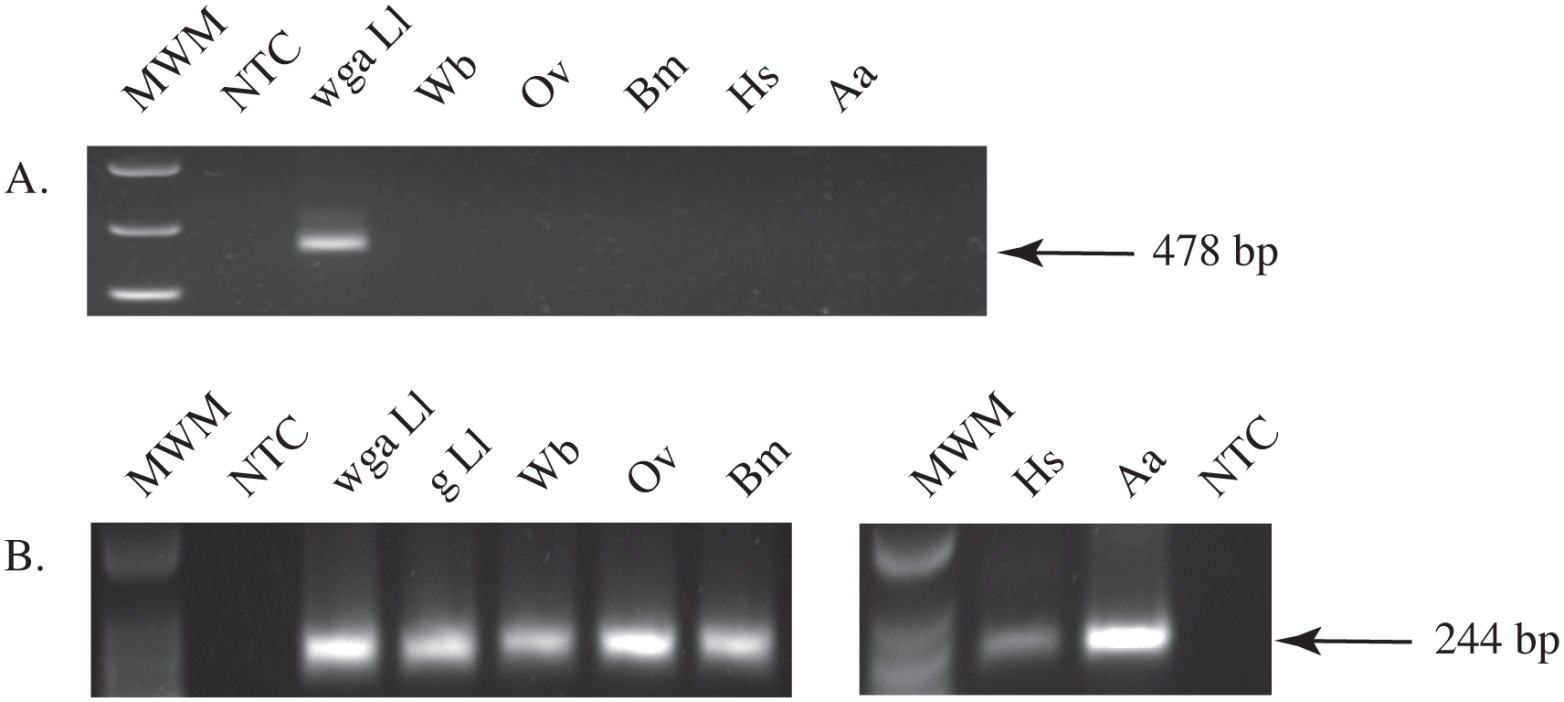
Species-specificity of RF4 as determined by PCR. Agarose gels showing specific amplification of *L*. *loa* RF4 **(A)** or a conserved 244 bp actin gene fragment **(B)** from WGA *L*. *loa* (WGA Ll), genomic *L*. *loa* (g Ll), *W*. *bancrofti* (Wb), *O*. *volvulus* (Ov), *B*. *malayi* (Bm), *A*. *albopictus* (Aa), and *H*. *sapiens* (Hs) DNA. PCR **(A)** and low molecular weight **(B)** markers (MWM) were used (New England Biolabs).

The species-specificity of RF4 was confirmed by PCR. A single band of the expected size (~480 bp) was amplified from *L*. *loa* but not from *W*. *bancrofti*, *O*. *volvulus*, *B*. *malayi*, *H*. *sapiens* or *A*. *albopictus* DNA (Fig 4A). The single band amplified from *L*. *loa* DNA indicates that RF4 is dispersed throughout the genome rather than organized in tandem arrays. The integrity of these DNA samples was confirmed in PCR experiments using primers designed to amplify a conserved actin gene. A single amplification product of 244 bp, the expected fragment size was obtained (Fig 4B).

**Fig 5:**
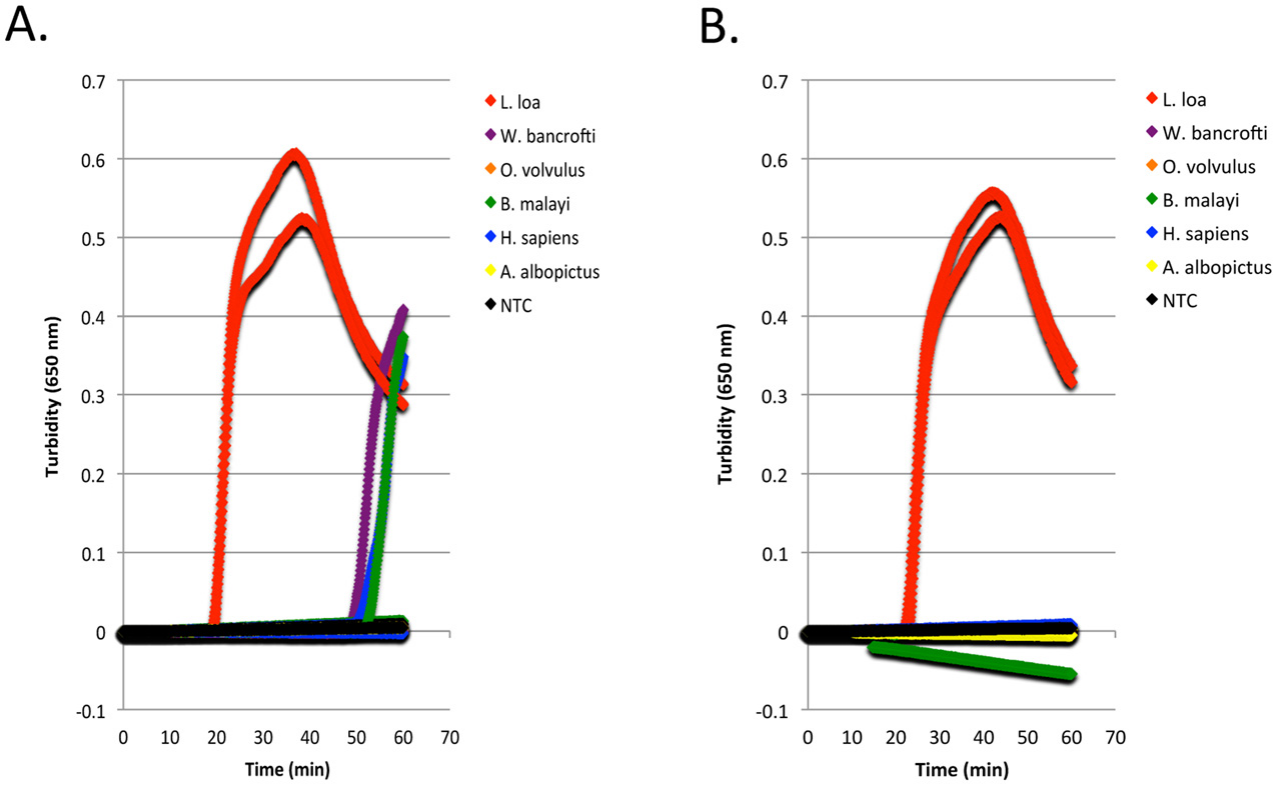
Species-specificity of the *L*. *loa* RF4-based LAMP assay. Turbidity curves generated with various DNAs (200 pg) amplified in the absence **(A)** or presence **(B)** of the V/DEF additive using the *L*. *loa* LAMP primer set with *Bst* 2.0. Each graph shows the results of two experiments.

The *L*. *loa* RF4 RepeatScout consensus sequence generated by aligning 326 sequences (Fig 1A and S1 Fig) was submitted to Primer Explorer for LAMP primer design. Loop forward and back primers were designed manually (Fig 1B). The specificity of RF4 was evaluated in LAMP assays using various genomic DNAs. As part of the assay optimization process, amplification in the presence and absence of the chemical additives, Valeramide and N,N,-diethylformamide was evaluated as background amplification occasionally occurs. Turbidity reached a threshold value of 0.1 in approximately 20 minutes when 0.2 ng *L*. *loa* DNA was added to reactions in the absence of additive (Fig 5A) compared to approximately 25 minutes in the presence of additive (Fig 5B). However, in reactions lacking additive, turbidity exceeding a threshold of 0.1 could be detected in approximately 50–60 minutes in some of the heterologous DNA samples whereas in reactions containing additive, turbidity remained negative (Fig 5B). No amplification was observed in the additive-plus or additive-minus non-template controls.

### The *L*. *loa* RF4-based LAMP Assay is Sensitive

**Fig 6:**
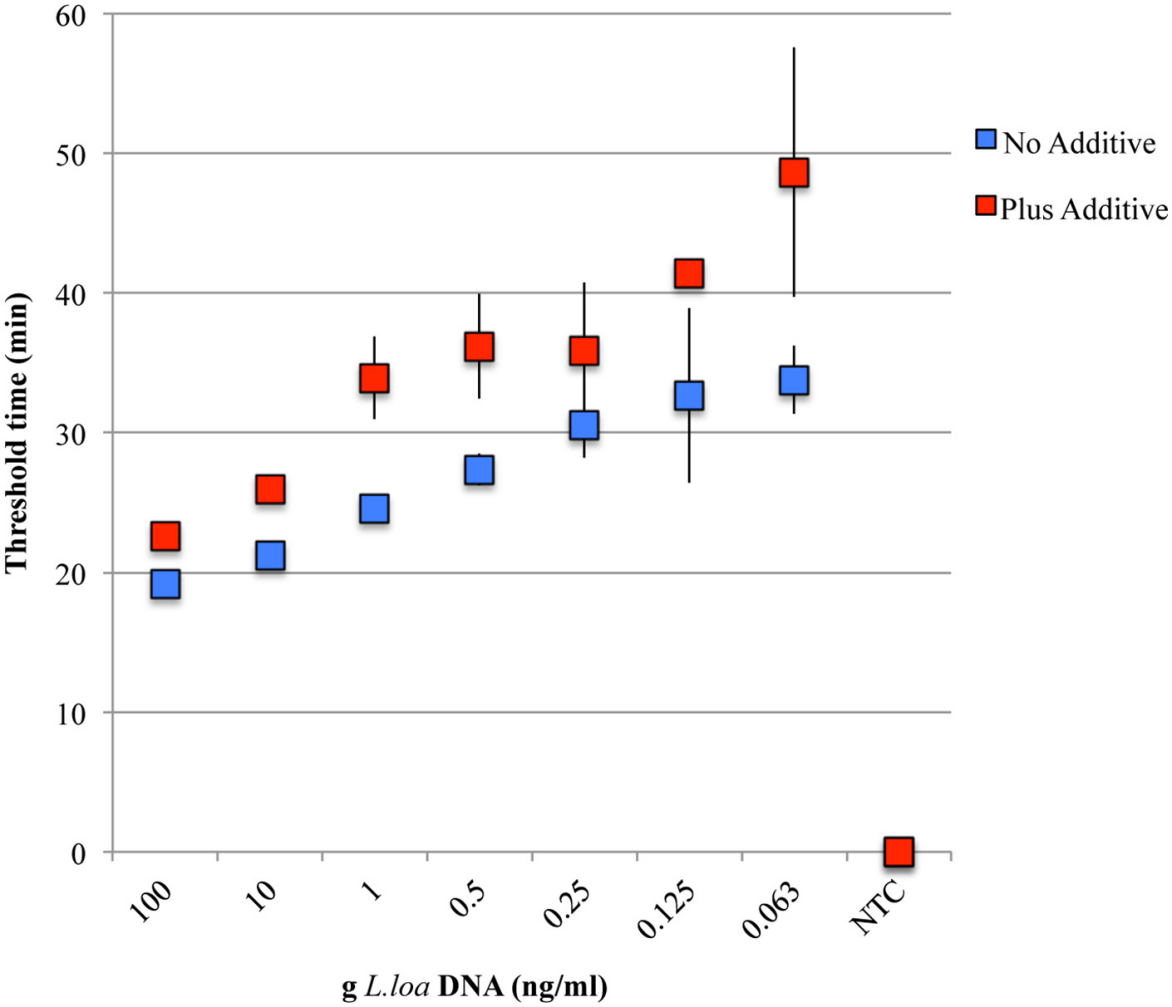
Sensitivity of the *L*. *loa* RF4-based LAMP assay. Dilutions of genomic *L*. *loa* DNA were amplified with the RF4 primer set and *Bst* 2.0 DNA polymerase in the absence (blue) or presence (red) of the V/DEF additive. Two ul of each dilution was added to LAMP reactions. The average threshold time, defined as the time at which the change in turbidity over time (dT/dt) reaches a value of 0.1, is plotted against the concentration of *L*. *loa* DNA (ng/ml). All reactions were performed in triplicate. Error bars represent the standard deviation at each point.

The sensitivity of the *L*. *loa* RF4 primer set was evaluated in the presence or absence of chemical additive (Fig 6). In the absence of additive, dilutions of genomic *L*. *loa* DNA amplified with the RF4 LAMP primer set reached a turbidity threshold of 0.1 ranging from 20 minutes for the highest concentration of template DNA (100 ng/ml) to ~ 35 minutes for the lowest. In the presence of additive, more time was required to reach the turbidity threshold of 0.1 for each DNA dilution. However, at the lowest concentration of DNA tested (0.063 ng/ml) turbidity reached the threshold of 0.1 in ~ 50 minutes; well within the cutoff time for the assay (60 minutes). At the lower concentrations of DNA (≤ 0.125 ng/ml), not all triplicates amplified. This was observed in reactions irrespective of additive status. No turbidity was observed in the NTCs regardless of the presence or absence of chemical additive.

**Fig 7:**
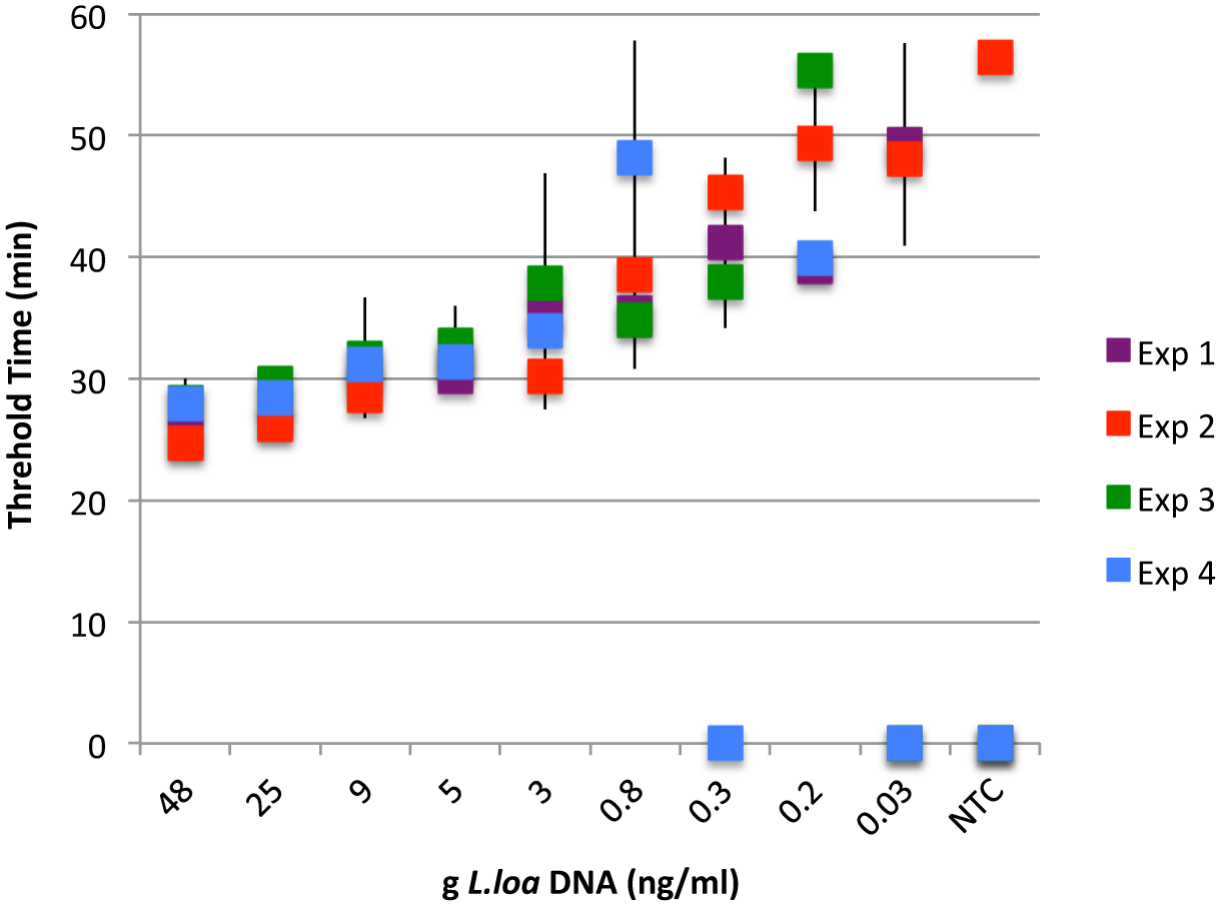
Detection of *L*. *loa* DNA in spiked blood samples. A two-fold dilution series of genomic *L*. *loa* DNA was prepared using uninfected human whole blood. NTCs only contained uninfected human whole blood. After DNA isolation, two μl of each dilution (or NTC) was used in LAMP reactions containing the V/DEF additive with *Bst* 2.0 DNA polymerase. For each experiment, all samples were assayed in triplicate. Average threshold times and standard deviations are plotted against ng DNA/ml of elution buffer.

To mimic a clinical situation, assays were performed on DNA extracted from a two-fold dilution series of genomic *L*. *loa* DNA spiked blood samples (Fig 7). Following extraction, the concentration of *L*. *loa* DNA in the samples ranged from ~ 0.03–50 ng/ml which is equivalent to 0.3–500 mf/ml blood. Three experiments were performed using 2 μl of each DNA dilution, equivalent to ~ 1-1/1600^th^ of an mf per LAMP reaction. Robust amplification was observed in samples containing equivalent amounts of template DNA down to 0.8 ng/ml of parasite DNA. Below this dilution, concordance between experiments was somewhat less but *L*. *loa* DNA was still easily detected within 60 minutes. The turbidity threshold of 0.1 was reached in 25–30 minutes for 100 pg with slightly more time required as the concentration of template DNA decreased (Fig 7). Turbidity was observed in only 1 of 12 NTCs tested over the course of the 4 experiments.

## Discussion

The recent availability of genome sequence from *L*. *loa* [43, 46] has enabled the development of a multi-step bioinformatic pipeline to perform a genome wide search for new diagnostic candidates. We have taken advantage of sequence information from the closely related filarial species *B*. *malayi*, *W*. *bancrofti* and *O*. *volvulus* as well as from human to identify species-specific repeats in the *L*. *loa* genome. We focused our efforts on repeat families with a copy number greater than or equal to 51 to maximize assay sensitivity and customized the process to select sequences ideally suited for LAMP, although the candidates were evaluated using both PCR and LAMP. The filarial genomes differ in repeat content with the *L*. *loa* genome being less repetitive (9.3%) than that of *B*. *malayi* (12.1%), but more repetitive than that of *W*. *bancrofti* (6.2%) [43].

We have previously developed a LAMP diagnostic assay for brugian filariasis using the *Brugia Hha I* repeat as the biomarker [39]. This amplification system was shown to be extremely sensitive in detecting *Brugia* DNA [39], with levels of sensitivity comparable to the *Hha I* PCR amplification system [48, 49]. Several repeat families have been employed as biomarkers, including the *Ssp I* repeat, to detect *W*. *bancrofti* in humans and vectors [50–53]. The *W*. *bancrofti* genome is estimated to contain more than 2000 copies of the *Ssp I* repeat, comprising ~0.5% of the genome [43]. Recently a LAMP assay based on *Ssp I* capable of detecting the equivalent of 1/1000^th^ of the DNA amount contained in a single microfilaria was published [54].

Using our genome filtering method, we identified ~9000 species-specific repeat families, ~300 of which are present with a copy number of 51 or more in the genome, and of these, 137 are of potential diagnostic value. Interestingly, the previously identified *L*. *loa* repeat LL3M9 used in both PCR [2] and LAMP assays [33] was filtered out in the process since orthologs were found in *B*. *malayi* and *W*. *bancrofti*. Our bioinformatic pipeline did not select the other *L*. *loa* sequence used as a LAMP biomarker, namely LLMF72, because it is a single copy gene. Of the six diagnostic candidates we validated in PCR and LAMP amplification studies, all were found to be species-specific with RF4 showing the most promise. Our analysis revealed RF4 to be part of a *Bel/Pao* LTR retroelement family present in metazoan genomes [47, 55]. This is consistent with its organization in the genome. This family was originally characterized with the discovery of element sequences such as *Pao* [56], *Bel* [57], *Tas* [58] and the various *Cer*-like sequences described in *Caenorhabditis elegans* [59]. The most prominent repeats in the *L*. *loa* and *W*. *bancrofti* genomes are the BEL retrotransposons that comprise 1.3% and 1.5% of the genome respectively [43]. To be of diagnostic value it is imperative that the DNA biomarker be stable and highly conserved in different geographic isolates. RepeatScout identified ~350 sequences related to RF4 in each of the two drafts available of the *L*. *loa* genome prepared from parasite isolates collected from different geographical locations namely Cameroon [43] and the Central African Republic [46] supporting its use in diagnostic tests. The RF4 consensus sequence generated by Repeat Scout is 440 bp long (33% GC).

High levels of specificity were achieved in RF4-based LAMP and PCR assays. LAMP primers amplified *L*. *loa* DNA, but not DNA isolated from the closely related filarial parasites *O*. *volvulus*, *B*. *malayi* or *W*.*bancrofti*, or from human or insect. Specificity was significantly enhanced, with negligible effect on sensitivity, in the presence of the chemical additives Valeramide and N,N,-diethylformamide. Combinations of small amides are thought to destabilize DNA secondary structure while leaving proper primer annealing alone [41, 60]. A similar specificity profile was obtained in PCR reactions, highlighting the versatility of this target for molecular diagnostic studies.

When highly purified DNA was used as template, LAMP amplification of RF4 was evident within 60 minutes at the lowest concentration of DNA tested (0.063 ng/ml; 2 μl is equivalent to 1/800 ^th^ of an mf), and most robust at 0.25 ng/ml and above. When LAMP was performed on varying amounts of *L*. *loa* DNA purified from spiked blood samples, robust amplification was observed in reactions containing 2 μl of 0.8 ng/ml or more parasite DNA. When the LAMP F3 and B3 primers were used to PCR amplify DNA from the spiked blood samples, 5 pg (1/20^th^ of mf) of starting material could be detected on agarose gels (data not shown). Therefore, RF4 can be used in both LAMP and PCR platforms, although LAMP provides greater sensitivity.

There are multiple reasons why LAMP has been adopted as a diagnostic platform to detect infectious agents including those responsible for neglected tropical diseases [19]. The main advantages of LAMP over PCR include its operational simplicity and isothermal nature. In PCR, thermal cycling is required to denature the template, anneal primers and extend the amplicon. LAMP employs Bst DNA polymerase, which provides both strand displacement and target amplification at a single temperature in a simple heat block or water bath at 60–65°C [30, 31]. Rapidity and versatility in readout options also make LAMP a particularly appealing technology. Cost is another factor with a recent estimate for a *W*. *bancrofti* LAMP test being $0.82 compared with more than $2.20 for PCR [54, 61]. In the present study, real-time turbidity was used for assay design and optimization yielding positive results within 60 minutes. However, in future studies we will use the more field-friendly hydroxy naphthol blue [62] and recently developed pH sensitive dyes [32] for detection.

In summary, we describe a LAMP diagnostic assay for *L*. *loa* based on a new DNA biomarker RF4 that generates a robust read-out within 60 minutes. The assay shows promise as a field tool for implementation and management of MDA programs and warrants further testing on clinical samples as the next stage in development towards this goal. Furthermore, we have successfully devised a method to mine the genome of *L*. *loa* for new biomarkers. We evaluated only a few repeat families resulting from our bioinformatics pipeline for their potential as diagnostic targets. While RF4 looks particularly promising, the bioinformatic output can be further analyzed to determine if more sensitive targets exist for LAMP and other amplification platforms. This comparative genomic approach can be applied to identify new diagnostic candidates for other filarial diseases and/or used to improve the sensitivity and specificity of existing molecular diagnostic methods.

## Supporting Information

S1 Fig Alignment of *L*. *loa* RF4 sequences. Sequences belonging to RF4 were identified in the *L*. *loa* genome and aligned by RepeatScout. The RF4 consensus sequence used for PCR and LAMP primer design is shown above the alignment. Within each supercontig containing an RF4 member, the location of the repeat is denoted in the sequence ID. Using the first sequence ID in the alignment as an example (Supercontig_3.5529_249_346/1-97), a 97 bp partial repeat is located between nucleotides 249–346 of supercontig_3.5529.
(TIF)

S2 Fig RF4 maps to the LTRs of BEL/PAO retrotransposons in *L*. *loa*. A) Diagram showing the location and orientation of the LTRs (black arrows) and organization of the GAG polyprotein (GAG), aspartic protease (AP), reverse transcriptase (RT), Ribonuclease H (Rnase H) and intregase (INT) of the BEL/PAO retrotransposon within the 12.3 Kb *L*. *loa* scaffold, 7180000007063_1 (GenBank acc.#JPEI01001237.1). B) MAP and alignment of the 600 bp *L*. *loa* LTR with the RF4 consensus sequence and the region spanning the LAMP primers. RepeatScout excluded the region of the *L*. *loa* LTR extending from bp 2–70 from the RF4 consensus due to homology with the *B*. *malayi* and *W*. *bancrofti* genomes. The region extending from bp 150–230 were excluded from RF4 because of homology with the *W*. *bancrofti* and *O*. *volvulus* genomes.
(TIF)

S1 Text List of the 137 RepeatScout consensus sequences identified by the bioinformatic pipeline. The consensus sequences are in fasta format. A descriptor of each consensus sequence follows the ">" symbol. It consists of a numerical name, copy number, %GC and length of the consensus repeat. Using the descriptor of the first consensus sequence in the list as an example (>R = 0_774_0_0_0_CG_35_bps_550), the name of this sequence is repeat 0. 774 copies of this family were identified by RepeatScout in the *L*. *loa* genome. The triplicate zeros indicate that no copies of this family were identified in the *O*. *volvulus*, *B*. *malayi* or *W*. *bancrofti* genomes. This consensus sequence is 35% GC rich and 550 bps long.
(DOCX)
