# Supplementary figures and images for: Genome Filtering for New DNA Biomarkers of Loa loa Infection Suitable for Loop-Mediated Isothermal Amplification
Source: PLoS One. 2015 Sep 28;10(9):e0139286. doi: 10.1371/journal.pone.0139286 (PMC4586141; doi:10.1371/journal.pone.0139286)

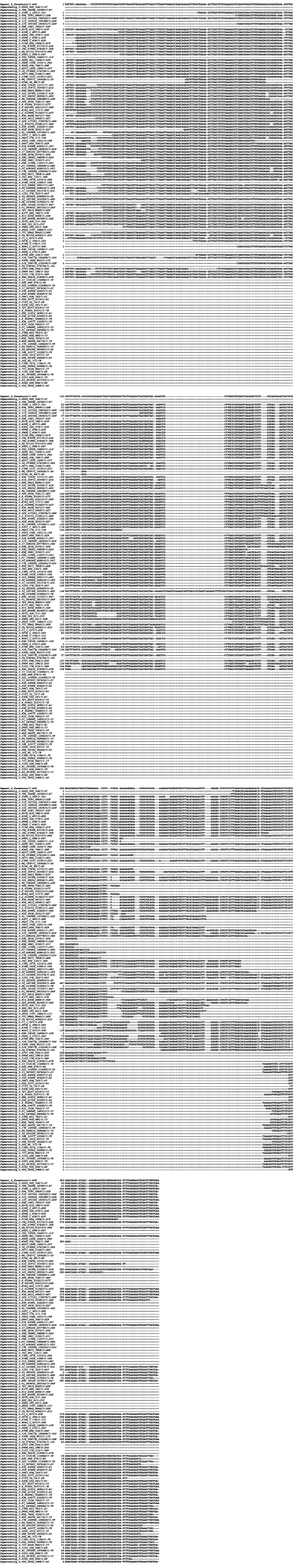

Supplement: S1 Fig — Sequences belonging to RF4 were identified in the L. loa genome and aligned by RepeatScout. The RF4 consensus sequence used for PCR and LAMP primer design is shown above the alignment. Within each supercontig containing an RF4 member, the location of the repeat is denoted in the sequence ID. Using the first sequence ID in the alignment as an example (Supercontig_3.5529_249_346/1-97), a 97 bp partial repeat is located between nucleotides 249–346 of supercontig_3.5529. (TIF) [file pone.0139286.s001.tif]

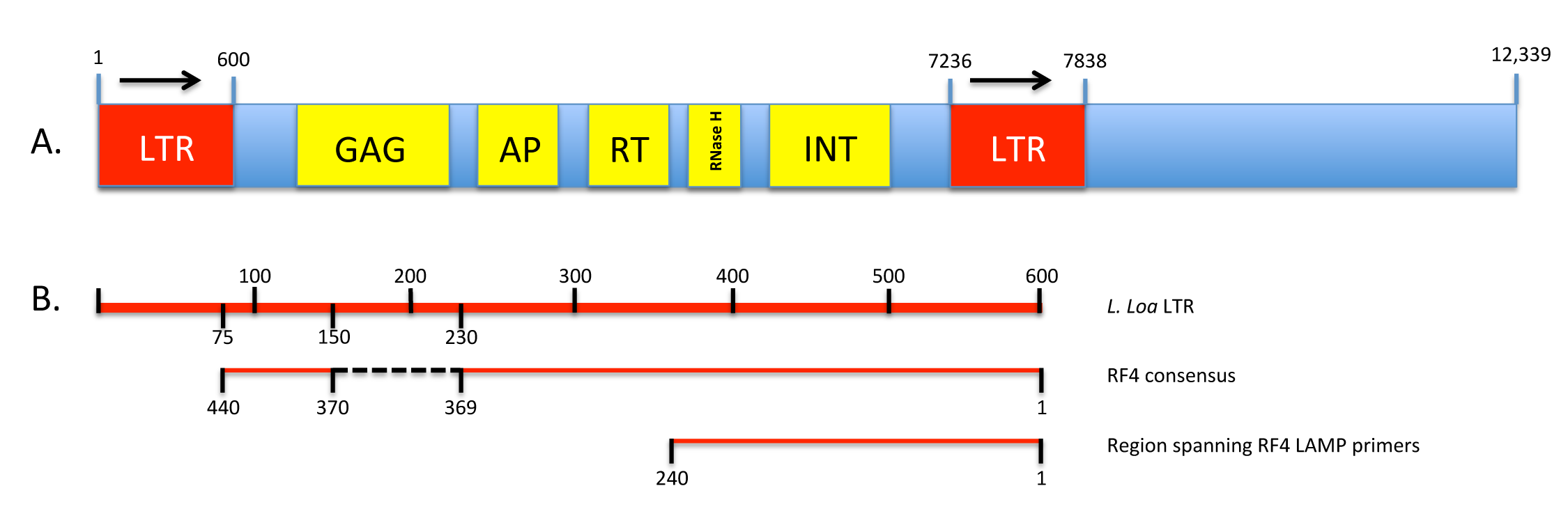

Supplement: S2 Fig — A) Diagram showing the location and orientation of the LTRs (black arrows) and organization of the GAG polyprotein (GAG), aspartic protease (AP), reverse transcriptase (RT), Ribonuclease H (Rnase H) and intregase (INT) of the BEL/PAO retrotransposon within the 12.3 Kb L. loa scaffold, 7180000007063_1 (GenBank acc.#JPEI01001237.1). B) MAP and alignment of the 600 bp L. loa LTR with the RF4 consensus sequence and the region spanning the LAMP primers. RepeatScout excluded the region of the L. loa LTR extending from bp 2–70 from the RF4 consensus due to homology with the B. malayi and W. bancrofti genomes. The region extending from bp 150–230 were excluded from RF4 because of homology with the W. bancrofti and O. volvulus genomes. (TIF) [file pone.0139286.s002.tif]
