# Supplementary material for: Genome Filtering for New DNA Biomarkers of Loa loa Infection Suitable for Loop-Mediated Isothermal Amplification
Source: PLoS One. 2015 Sep 28;10(9):e0139286. doi: 10.1371/journal.pone.0139286 (PMC4586141; doi:10.1371/journal.pone.0139286)
Supplement: S1 Text — The consensus sequences are in fasta format. A descriptor of each consensus sequence follows the ">" symbol. It consists of a numerical name, copy number, %GC and length of the consensus repeat. Using the descriptor of the first consensus sequence in the list as an example (>R = 0_774_0_0_0_CG_35_bps_550), the name of this sequence is repeat 0. 774 copies of this family were identified by RepeatScout in the L. loa genome. The triplicate zeros indicate that no copies of this family were identified in the O. volvulus, B. malayi or W. bancrofti genomes. This consensus sequence is 35% GC rich and 550 bps long. (DOCX) [file pone.0139286.s003.docx]

>R=0_774_0_0_0_CG_35_bps_550

TTTGGAAATTGTTTTTCTGTTGAACTCGTTTCTCGGCGATACAGATTTATTCGGGATGTAATATTGTTCTTGCACAATATTACTAGTTAACAACAAGAAAAAGGTAAAAAATATATATGTATATAACACGATGAATTGGTAAGCAATGATAAATGAGTTATACAAAAAGCAAGTAATATGATCAACTAACAAACTCCTTGTCGTTGCCTTGGATAATAATTTGACTGTCGGTTAGTTGATAGGTCGTTGAGTTGGGAATATGTTACGGGAAATGTATTTAGCGAATAGAAGAATATCGTAGTTTGTTGTCCAATGGTGGCGGATATGCGAAGATGTCGTTGTTTGTGGTTTAGTCGTGGAGGATAAGCGATGAGGATGTATCGTCTGCTCGATTGTAAAAACTAACTCAACTGTCTGTCTCGAGATTTTTTTATATATATAGAAGGATTAGGGAGAATTTTATAAGCTCCATCGGGGATATAATGGAGGTATGGCTGGCCTTTGGCATTGTCCATTGTGTAGGTACAATGAATTTTTTGTTTTATGTTGT

>R=3_306_0_0_0_CG_27_bps_679

AAAAATTATGAGAATGAGAAGATGAGGATTACTGTTCTTGTTAGTAATAAAACAGTTTTTTTTTTTTAAAAAAATGACTTACTGAATACTGATTGGTTATGTGTTCGAGGAGTATAATAGTTATCCTTTATTGTTGTTCAAATAACAAATACAATAAAAATAGGTTAGAAGTAATTCCGATTATAGTAGAATGAAGTACAAATTAATAACGTAGAAAAAAGGGAGAAATATAGAAGTAACAATTCATAAATGGAGAGGAAATAAATCAGGATAGTGTTGTGTAGAGTGAAGTGGAGAAAGATAAAAATTAACATTTAATCTTGTTAGAGATTCTTAGGGTTTATATATGGGATGTGTGAAGAGGAACAATCATTACATATTGACAGTATTCTCACAGGTGCAAGAGAAATTTACATATAAGAACAGTTTTCTTTAGAAAAAATTGCATAGAAATTATTTTATCTGATGTACAGCTTTCTCACCATTGTATAAAAGATTTTGCATGGGATAAAGCCTTTACAAATTAGTTACCATATGTGCACAAATCTTAACATCAAAAAAAGCACTTTTAGTAGATTAGATAACTGACTCATGACTTTAAGCATGAGAGAAAAAATATGTAAATGCGATCTATTTGAATGAATGATTTTACGATATAACTATTTCGGTTTTTCCGACA

>R=4_368_0_0_0_CG_33_bps_440

TGTTGTGAAAAAATTCTTTCTTTTTATCGAGTCGTTTAGATTTAGAAGGTTTAGCCTTGAGTTAGGATCAAGCAGAAATTTAATTAAAGGCTTACCTTGTGAAGACGTCGAATATACGTTGTGAGGACACAGAGTAAAATTTACCGCTTTATTGATATAATACAGAGTTGATCAGTAGGATTATTGAAGAATAACGAGTAGAAAATCGCTTGACCGTGAGTCAAAGGCTGTTGTAAGTAAACTACTATGGAAGAGCACTAGTCCAGGAAAACCTTTTTACAAAAAGGGAATATATATATAAAGAAGTAGGAGTGGTTTACATAAAACGTTAGAGGGTATTCCTTTAGATACAAAGGAAACGGTAAAAATGTGTATTTCCGTTAGACAAAGATAACAAAGAAACGTATGGGAGATAATTTTGAGGAATGTGATTTGGTGAA

>R=8_182_0_0_0_CG_28_bps_430

GTCGTAAAAACCGAAATAATTGTATCATGAAATTATTCATTCAAATAGTTTCCATTTACATTTGTTTTTTCTCTCTCATGCCTAAGGTCATGAGTCAGCTATCTAATCTTTAAATCGTCTAAATGAGCTTTATCTGATGTTGAGATTTGCGCACATCTGGATGACAACGAATCTTATAAACATGGATGGGCATACTGTAGAGATATCTAATCTTATAAACATGTTTATAAGATACATATTTATCCATAAATCTCTGTTAAGTATAGAATTTTTTTCCTTTCTCCATACGTACTCAATTCATAAACATAAGAAAAACTTTAAATTAATCATTGATATAAAAATTACTTCAGTCGCTAACTCAAGATAAAGAAAAAACAACTGTATCTACTAACCCTTTCAATGAATAATTTCATAATACAATTATTTCGGT

>R=9_250_0_0_0_CG_32_bps_398

ACGAATATTCTGAGAAGGTATTCTTCGTTTGGTTCAGGAGGAAGTTATTAATTCGAAGAGGATAGCAGGTTATAATAACGCAGAGGATAGAAGTAAAATATTTATTGAAGAGAGTATAGGATACAATGTACGGTGCAGTCCTTGGTTGAGTGTTTGAAAAGAAAAGATGATCTTCCAGTTATAGCCTTGAGTATATATACAATTTTAATTAACACAATGAGCATATTTGGGGGGATTGGTTTATAACAAACATTACATCAGATTTTCCTTTGGATAAAGAAAAACTGTCAAAATCCGATTACAACAAAAGGCTCTGTTTGCAACATTAAGCATTGCAATTAAGATTGGTTCTTAGACAAAGAAATTTAGCTTATGAGTGTGCTTTGTTACATAAAAAA

>R=10_140_0_0_0_CG_34_bps_375

TGTCGTGAGAAAATAAAAGAAAGGGAGTTAAGAAGGTGAGGTAGATTGTTATTGTTCGTTGTAGAAGCTGTTATAATTTTAAAGGAATGACTTACAGTGTATCGGTTCGTTTTGTGTTCGAAACTTACAACAGATTATCCTTTATTATCGTCTGGATACCAAATACAGTAAAGACAGGTTCAAATAATTCCAATTACAATAGAATGACTTCCAGGGTAAAGCCAGTAGAATAATGAGATTTTGTAAGGATTAGTAGATTGGAAAGTGTATACGTAGACTGGTGTGTGGACCTGAGTGTCGATAGATGGAATTTTAGAAACTATTAGGATTTCTTAGGTTTTTATAAGGAGTGTGTAAGGAGAAAGTAAGGAGAAA

>R=14_142_0_0_0_CG_33_bps_565

CAATGAAGTGATATCTAAGCAATGTTCATTCTAGACCCAGGGGAAGGAAATCCACAGTTGGAACATACTTTGGCAGACACACGAAACCATCACACTGCTGGTCAAAGTTCAGCAAGTCAACAAGATCGCTCGAATGATGAGTATCCCTCAGAAAAATTTAACATCTTGGACTATCCCGATGATGACATCTTCAACTATCTTAACATCCCCGACGATGTTGTTGTTAATATCGAAGATATTAACATCGACAACTCTAATGGTAATATCTTCGAATGTTAGCATCGCCGACGATGGCAACTTCCTCGACGACTTCAAAATATTTTGCCATCAATGAAAATTGTCTGCTTGCAGTATATCAAAACTATTTATCCCTTCTCTCAGTATATAAGTTTTATAGTAAGAAAAAAAATTTTGGTTTAAAAATAAAATTTTTTTTTTTACCTTGGGAGAATTATCTAAGGATTTTATATAAAAAGAATATATAAAAAAATTTTCCATTGGAGCTTTGAGTACATTCGACGAATGCTTGCACTTCTTGGCAAAATTTCAGAAATATCAAAAATAT

>R=15_148_0_0_0_CG_31_bps_437

TTATTCTCATATTGTAATCCACACCCACTTGTTCTCAATATAAATTAACAAGGATTTGAGAATAGTACGCACTATTGACTCAATAAACACTATATACGCATTCATTCCTGCAAAGGAATCTTTACTACTTCACTTAAATCACCGATCCAACTATATTTTGCTCTGTTTCTGTTTTATCCTCCAATAATTCATTTCAATCTATTCCTCAGAATTAAAGTAACGATTGTGTCTCATTTGCGTTAATTTCTATTTAGTTAATAAATACTATCTGTATTCCTGGTATTCAGCTACATCAAAAGGATCAACAGATCCTCACCCATTCATTGATAATTCAATCTTAACAAAACAATTTCTCTTAACTAAACGTGGAAAACAAGCTTTCTTTCAATAAAGGAAGCAAGGAAGTCGAGTTTTATTTGTTACGAAAGAAAAAAACC

>R=16_232_0_0_0_CG_31_bps_494

ACGGTAAATGATTAGTAAAGATTTAGACCCAAATTCGGTTGCTTCAATGAAGGATAGACTGTTTTAGCTACTATGACTAATCAAGACTATGAAATGAGGAGAAGAAATTGTAGAAACGAATTTGGTGAGGAAGAAAGCAGTTTGTGACGGTAGACTGAATTCTGAAGGAAAATATGGACCTTTTACAGTGAAAAGATCGAATCAGTCAAATATCAGAAAGAATCAAGAAAAATCGAAATAAATCTCAGTTTTCCATTTTGCATTGAATAACAATGCATTGTAAAACAGAAAGTATTTCATACAGATTATGCAAAAAAGTGAATAATACTGTCACAAACAGTTTTTCTATAGAAATTGGGATTAACAGAATATTTCGTGTCACTGGAAGCCTTTCATTGAATTACATAAATTTGCATTTGTGTCAGAAATAGAAGTAACCAAAAGTATGCTGTACCATCGATTGTCATTATTTTCCATTCAGGGAAAGTTGAATT

>R=17_118_0_0_0_CG_31_bps_676

TAAAGTTTGAATTTGCATCATTACGTCATCAAACCTTCGTCTCTCATAAGTTCTTTCTGATTTTTAGTAAATTATTATTCTTGACAGAAACAAAGAATGAAACATGCCCCAAAATCGGTGAAATTCGTCTTAGATACGAGTTCCAAAGCTTTCTTCAAATATGGCGGTGTTATTTTATCGTCCCGGTAATGTAATATTCTATATCGACGGTTCAACTCATAGTTTGCACATTCAGACATCTCGGGATTCCCCTGTAAGATGATATAAAATAATAATCAATCATTATTATTACCAATCAATTCATTATAACTAATTAACTCATTTCAATTTCCCCTATCTCCTACAGTTACTAGTAATTGAAATTACAAAATTTAGCGGATATGATTTTTCAACCTTAGGGAATATCTGTAGCGTGAGAATATATAAATGAAAAAAAAGAGCGCACATGCTAAGATTTTTTAAATCATCGCTGGGTAAGTAACCAACAATTAGATGTCCCACTTCTCCAAAAACAGAGACTACTTTATCGTTGGGATACCGAAATTTAATTTCAATCTTTCTTTTTTTGTGAGCGCTGGGTCCTTCCTCATCTGTTCCTCCTGAAATTTTAATTAATTCATCATTAAAGTTCTTGTTCTTTATATATTTAAACAGAAAATGTAAATTCATTCAAATT

>R=19_324_0_0_0_CG_29_bps_314

TAAATTAATAAAATAAATAATTAAATATATAAAGTAATGAATTATTCATCCAAATCCAACTCTTCCAATTTTTTAACCAATTTTAATTGCAATGTTTAAATGTTGCAGACAAAGCCCTTTGTTGTAAACTTTTTCCAATCTGTTAATCAAGCTTACATGCAATTGTCGGAATGTTGTAAAGAACTTATATGCAAATATCGAAATCTTATAAAGAACCTCTAGTCTGGTAGTTAAACTAATGTGTCTGAAATGTTAAACCACTCCCACTATAAAAGGAGCTGGGATCAATCATCCGGACGATCAGTTACTAAATC

>R=21_106_0_0_0_CG_26_bps_376

TTTTTTCCCCCAAAAGAGGCAGTGTGCATTTCGGTGGGAAGGAATAATTAAATCATTTTAGGTACAAAATGATTTAACTAAAAGAAATTGAATCCTTGACACTAAGTTCAATATTTGATACGAAAAATATTTTTCTTTTTGGTAATTTCATTGCTTCCAAAACCGATGTAATTTGCAGTACTGTCCATCACTATTTAAGTTCCAGTTAAAAAAAAATTTTGATTAAAGAGCAAAAGTGAACATATAATTTAAATATTGATCTTCCTACAGTAATAGTTGATATGAGATTAATCAGCGTAATTCTACTGAAAATGATATTTGTTTCTTAAATTTGAATAGATGAATAGTCTCATACATTTTGTATTTGCTTATTTAT

>R=22_114_0_0_0_CG_26_bps_577

TTTTTTTTTTTTTTGGATTTCCTTGCACGGATTTCTATTTAGCAGAAGCTGTAATTAACAGTAGGAATTTATATGATTTCTGCTATTAAACTTATCTTGTTTTTTTACGACGTTATAGTGTACATATTTGTAAATTTTCGGCTGTATTTGAATTCAGTTTTTCTGCGCCTCAAATTTTCAGTTTCTTGAAATACAGAAAAAAGATTTATTAATGACTATAAGATTATTTAAAAAAATTGCAGACTAGACTGTTTCAAACCATTATATTGTTTGACCGGATAGATAGTTCAAAATGATTTTACAGAAACCCGATGCATTTCAGTTTATTTTTGACCAACATTATATATAAATAAGCAAATACAAAATGTATGAGACTATTCATCTATTCAAATTTAAGAAACAAACAACATTTTCAGTAGAATTACGCTGATTAATCTCATATCAACTATTACTGTAGGAACATCAATATTTAAATTATATGTTCACTTTTGCTCTTTAATCAAAATTTTTTTTTAACTGGAACTTAAATAGTGATGGACAGTACTGCAAATTACATCGGTTTTGGAAGCAATGAAAT

>R=23_128_0_0_0_CG_30_bps_403

TATAAACGACAAAATTTAAACCCTGCTGAAATAAAAACATTTTAATTACTGAATTGAAATACATATAAATCCGTGTAAAAAATTTTTTGTCAACAGATGTCGCGAATAAACGGAAAAAAATTCGTGAAAAAGAATGAGGAATGTGTAGAGCACGTATTAATTTGCATATACCATGTTAAATCATGAGTTTTTTCTGTATGAATGTGATGTTATCACTGCATAGAATTGTACATGTGCTCATTGTTTAGCAATGTACCCAAAGAGTCTTACACTAATGTGCGCTAACAAAGAGTGCTTTATCAGTATGAGCTAACAAAGGTGCTAATCTTAGTAATCGTAAAATCATTCAATCCTGCTAATAGTTACGCAAAAAATTTTCAAGAATACAAAAATTTTCCACTTT

>R=24_164_0_0_0_CG_31_bps_577

AATTCTAAGAATTTTAAGAATGGATCCAATCATTTTGGATGTGTAATAAATCTGGTTGCTAAAGGATGCAATTTTCCCCCGAGTAATTCGTAAAACAAATTTTTTCATTTCGCCACTTTATTGTATGCTTCTTTAGATGAGTAATGACAATAAATGATTAGTAATATATATGAGATTTCGGCCCAAATTCGGTCGCTCAGTGAAGGATAAGCTCTATAATTACTTTATGCATTAGTTACGATGACTAATCAATACTATGAAACGAGTAAAAGAAATTGTAGAAACGAATTTGGTGAGGAAGGAGGAAGTTTGTGACAGTAGACTGAATTCTGAACGAAAATATTGACCTTTTATAGTGAAAAAATCGAATCAGTCAAAGATCAGAAAGGTTCGGGGAAAATCGAAATTAATCTCAATTTTCCATTTAGCATTGAATAGCAAGGCATTGTAAAACAGAAAGCATTTCATACAGATTATACAAAAAAGTAAATAATACTGTCACATACAGTTTTTTTATGCAAACTGGGATTAACAAAATATTTTGTGTCACTGGAAGCCTTTCATGAATTGTCACAAA

>R=26_130_0_0_0_CG_30_bps_335

ATTAAAAGAATATTTCGTGTCACTGGAAGCCTTCCATTGAATTACATAAATTTGCATTTGTGTCAGAAATAGAAGCATCCAAAAGTATGCTGGACCATCGATTGTCATTATTTTCCATTCAGGGAAAGTTGTAGAATTTACATCATTTTCTATTATTTCTGCCTTTTGTATTTTAATATAAATTTGCAATCCTACAAGTCTTTCGAACTTCCAGAACGATGAATCGGAAATTTTTGTCCGAAACTGAAAAAAACAATCAGTATTCCGCTAAAAAAAAAATATGAAATTCAAGAGTAGAAAATGTATGATAATGATAAGTAAGTAACCTGCGATAA

>R=27_224_0_0_0_CG_34_bps_370

TAAATTTCTTTGTCTAAGAACCGATCTTAATTGCAATATGCTTGATGTTGCATACAGAGCCTTTTGTTGTAACCGGATTTTGACAGTTTTTCTTTATCCAAAGCAAAATATAATGTAATGTTTGTTCTAAACCACACCCCCCAAATATGCCCAATGTGTTAATTAAAATTGTTTATATACTCAAGACTATAACTACATCATTTTCTTTTTCAAACACTCAGCCAAGTACTGCAACGTACAATGTTTCCTCTACTATCCTCTGCGATATTTTTACTACTATCCTCTGCGAATTAATAACTTCCTCCTGAACCGAACGAAGAATACCTTCTCAGAATATTCGTCTGTGCCTAAGAACAAACCCTTCCAAATC

>28_104_0_0_0_CG_31_bps_444

TTTTTAAATTTTATGTAACAAATCATTTACTTGAGAAATTTACGAATTCTAATTAAAACCACTCATAACACTTTTTTTCAGCTTTCCCGACAGAAGCTGGTGGGTATGTTTGGGATATTTGAAAGAGCATGATCCACATCGTGTTTACAACCGAATGTAAAAATCATTATCTAATTGATTGGGCGGAATGGCTGATGCAGCTCTAACCGACAACAATCCAGAATGACAAATTAATTCCTTGATAACGGATTCACTAATGACATCAGATTTGTATAAGCAAGATTTAAAAGCAATTCTTTATCATTATAACAGACAAATTACTTCATCAAAGCATTTATCAAAACTGGTTAAAGATATTTTTGATATTTCCGAAATTTCGCCAAGAAGTGCAAGCATTCGTCGAAAGTATTCAAAGCTCCAATGGAAAATTTTTTAATATATTCT

>R=31_78_0_0_0_CG_28_bps_381

AATTCAATAATAAAAGCTGATTTAAATATTAATTTTTTGAAGTCTTTTGTAGTTGTTGTTTTTCTGAATATCATAAAATAGATCTGAGGTATTTTCAAAACACAATAAGTTCCAGAAAATACGTATGCCAAGTTTTCATTGAAACGATTTCTGAGAATATTAAACGATCAGACGGCAATCCACCTGATCGCTAAAAAAACGTAAATACAAAGTAATAACAAGCTGGAACATAGTCGCTTCACTCACAGTTCGAAAATTTATTAACCGAAAAAAAAAATAAGAGCATAATAATAAAAAGAGGAATTTTCTAAATTTCCACTTCCTGCACATTTTTCATTTCCAATCCTTAAGAAAATGTCATTGAGCGTTTAACTAAGACTC

> R=32_220_0_0_0_CG_31_bps_406

AAAATTGAAAGTAAAAGATTTTGATTTGAAAGGTTTTATTTTGAAGGATTTATTCTTAAGCATAGACGAATATTTTGAGAAGGTATTCCTCGTTCGGTTTAGGAGGAAGTTATTAATTTGCAGCGGATAGTAGTAAAATATTTATCAAAGAGAATAGAGGATACAATGCATGTTGCAGTCCTTGGCTGAGTGTTTGAAAAGGAAAATGATGTAGTTATAGCCTTGAGTATATATACAATTTTAATTAACACAGTGGGCATATTTGGGGGGTGTGGTTTAGAACAAACATTACATAAAATTATACTTCGGATAAAGAAAAACTGTCAAAATCCGATTACAACAAAAGGCTCTGTATGCAACATCAAGCATATTGCAATCAAGATCGGTTCTTAGACAAAGAAATTTA

>R=34_328_0_0_0_CG_35_bps_1068

AAATTTCGGTATCCCAATAATAAACTAGTCTCTGCTCTTGGACAAGTGGGACATATAATTGTTCGTCACTTGCCCCACAAAGATTTAAGAAATCTTAGCAAGTTAGCACTTTTCTTTCACTTATATATTATCACGCTACAGATATTCACCAAGTGTCGGGAAAGCTATTCTGTTGATCGTTGAACATTCGTTCATTGGATATTGTGGAAATTTGATTTGAACGTAGCTTTAATTTCGGCAATCCGACACCAACGTCAAAAAATCATGTCCGCCAAATTCTGTGATTACAATTACTAGTAACTGTAGGAGATAAGGGAAATTGAAATGAGTTAATAAGCTATAACGAAATGATTGTAAATAATGAAAATAAAGGAATTTTTTTTCATATCATCTTACAGGGCAATCCCCATGATGTCTGAGCATATAAACCACGAGTTAAACCGTCGACGTGGAATACTGCATTACCGAGATGGTGAAATAAACCCGCCATATTTAAAGAATGCTTTGAAAGGCATAGATGAGACGAATTTCACCGGTTTTCAGGCATGTTCCATTCTTTGTTCCTGTCAAGAATAATAATTTACTAAAAATCAGAAAGAACTTATAACAGACGATGATTTGACGACATAATGATGCAAATTCGAACTTTAGCACCAAGAACCCTTCGCGGTGCCTCGTTGGAAACCACATGAAGCTATTTTGTTTGGAAGAGAAAAACCGATCAATATAACAGCCTCGGAGAACTGTTATTGTACAGTACTGATATTCACGTCTCAGGAACATCATCTAGAAATACGCAAGGAATTCGTAACTGGTTAGGAAATTGTAATGGATTTTTGCATCATATGGGATTAAACCACTATTAAACAAACAAATAGTTCTAATTATATGATTTCAGCAAATATGCCATTCCTCCCCAATGAAATAGAAGGAAGTCCAATGAACGGAGTGATTATCCCATACGATTTAAGTTCATCCTCGTACAAGAGATTTTCTTTCTGGCAAAACGACGATTTGTTAGTCATCTTTTATATTAACAGAAACAAACTTTAATCATTATCAAATTAT

>R=39_656_0_0_0_CG_33_bps_380

TGTCGGGAAAGCTAATCTGTTGAATTCGTTTTTCAGCGATATAGATTTATTCGGGATGTAATATTGTTCTTGCACAATATTACTAGTTAACAACAAAAAGGGTGTAAAATATATATATATATAATATACGAAATTAGTAAGCAGTGATGAGTTATACAAAAAATAAGCAATATGATCAACTAACAAATTCCTTGTCGTTGCCTTCGATAATAATTTGACTGTCGGTTAGTTGATAGGTCGTTGAATTGGGAATATGTTACGGGAAATGTATTTAGCAAATAGTAAAAATATCGTAGTTTGTTGTCCAGTGGTGGCGGATATGCGAAGATGTCGTTGTAGTTCAGTCGTGGAGAATAAGCGTAGAGGATGTATTGTCTGCT

>R=41_54_0_0_0_CG_30_bps_302

CTTTTTTACGCTAAAAGTCACGAAATGCGCAATAGTTGTTTGTAGCATTTAAACATTTCCAGCTTGTCATTAACACAAATTAGCACAGCTTCGAAAGGAACAAACGGAAAATTAAGCATCAATAAGCAACCATAAATATACATGTGCTATCTGACGAATCAATAAATGTACGTTTGCAAATTGTACACTTCTCATTTGTTCGACCTAAAAAATCAATAAATCCACATTTGCATTTATTAATTTATCAAGACGGACATTTTCACTATCTAGAGATTCAACAAATGTATTTATCAATATTATTA

>R=45_242_0_0_0_CG_28_bps_1177

TTTGTGACAATGCATGAAAGGCTTCCAATGACACAAAATATTCTGTTAATCCCAATTTGCATAAAAAAACTGTATATGACAGTATTATTTACTTTTTTGCATAATCTGTATAAAATGCTTTCTGTTTTACAATGCCTTGCTATTCAATGCTAAATGGAAAATTGAGATTAATTTCGATTTTCCCCGAACCTTTCTGATCTTTGACTGATTCGATTTTTTCACTATAAAAGGTCAATATTTTCGTTCAGAATTCAGTCTACTGTCACAAACTGCCTCCCTCCTCACCAAATTCGTTTCTACAATTTCTTTTACTCGTTTCATAGTATTGATTAGTCATCGTAACTAATGCAAAAAGTAATTATAGAGCTTATCCTTCACTGAAGCAACCGAATTTGGGCGCAAATCTCATACATATTACTAATCATTTACTGTCATTACTCATCAAAAGAAGCATACAATAAAGTGGCGAAATGAAAAAATTTGTTTTACGAATTACTCGGAGGAAAATTGCATCCTTTAGCAACCAGATTTATTACACATTCAAAATGATGGAATCTATTCTCTAAAAAACTCGAAATTCACTTGTAATAGTAAAAAAATTCGACAATACCTTAAGATTATTCAAATCATTGAGTATATTTTTTATTTATACATATTTTCAAATAACAATGTATTTTAAATATCTTGCTATCCTTACATTCATCGTGATATCATTCGATATTACATCAGCAACAAGTAGACTTTTTGAATTTTCAAATTTTAAATTTAAATGAACTTTTTTAAAGTGGTACACTATACATAATTAGGAAATTTGAATGAATTTGCATTTTCTGTTTAAATATCTGAAAAACAAGAACTTTAGGATGAATTGAGGAAAGACCCAGCCCGCACAAAAAAAAAGGAACACAGAAACCAAAAAAAGAAATACCAAAAATAAAGTAACCTATCCCAATAAAAAAGTAGGAGATCAAATTGCACAAGACGGACCCATAATAGATCGAAAAAATCCCAGCAAGGAATCAATAAACCTTAACATACAAACAATCACCTTATACATATTTATTAACACGCAAAAAATATTCCCCAAATTTTATGATTTCAATTACTAGTAACTGTAGGAAATAAGGAAAATTGAAATGAGTTAATGAGTTAAAATGAAACGATTAATAATACTAAT

>53_248_0_0_0_CG_35_bps_461

CGAAACCTCAAATAAATTCATTCACATTGTAAATTAATGCTAATGATACTATGAATCACTCTTAACATATCTACATTCCTTAAAATTTTTTCCCACACTTTTTTGTTGTTATCTTATCTAACCAATTTGCATTTTTATGTTTAAACCCTTAATTTCCCATGTCAATCATTCTGTTTAATCTTGTTATAATGCTTGTACCATATCCGGTGTGTTTCCGATACTTGAATTCTTCATGTAAACCACTCCCATTTCTTAACATATATAATCTCGGTCCCAAATCCGGGGGGGGTTTTTGGCTGCTCTCCTCTTCGAAGGTTATCGGCTTCTTCTTCTGTTTATTCTTTGCTTCTACGGACATTGGCGGATTTTTATCAATCTCTTATCACGATAAAGGTTAATATCCCTTACTGTTGCGTATTCACAAGACAAATCGACCTCCGGACAAACGCTTTCGCACTAAG

>R=57_450_0_0_0_CG_34_bps_1446

CCATTCACTGAAAAAACTGTTGCAGTTAAAGACAAACATGACAATGATCAATATACCCATTGAATATTAAATATTCAGTAGAGTTTTGTATTGATCTTTCGGGGCAGAATGAATTACATTCTTGGTATATTGGTTCAATTCTAATTCTAATTGCATTCCTTAATTAAATTGTTTCCGCTATTATGCCTTCTGCTTACAATTCTTAGTAAATTTAGACAAAGAGAGATTTCATTATCACCCAAGTATCACTTCATTATCATTATCACTCAAATATTATGACCATTCGACTATCACTTATAATTGATGCAAAGTTTGGAATAGAAATCACTGCAGTATTTGTACCAAAAACGATTAAATCTCAACATTGGATGATTTTGGCATAATTTGATAATGATTAAAGTTTGCTTCTGTTAATATAAAAGATGACTAACAAATCGTCGGCTTGCCAGAAAGAAAATCTCTTGTACGAGGATGAACTTAAATCGTATGGGATAATCACTCCGTTCATTGGACTTCCTTCTATTTCATTGGGGAGGAATGGCATATCTGCTGAAATCATATAATTAGAACTATTTGTTTGTTTAATAGTGGTTTAATCCCATATGATACAAAAATCCATTACAATTTCCTAACCAGTTACGAATTCCCTGCGTATTTCTAGATGATGTTCCTGAGACGTGAATATCAGTACTGTACAATAACAGTTATCCGAGGCTGTTATATTGATCGGTTTTTCACTTCCAAACAAAATAGCTTCATGTGGTTTCCAAAAAGGCACCGCGAAGGGTTCTTGGTGCTAAAGTTCGAATTTGCATCATTATGTCGTCAAATCATCGTCTGTTATAAGTTCTTTCTGATTTTTAGTAAATTATTATTCTTGACAGGAACAAAGAATGGAACATGCCTGAAAACCGGTGAAATTCGTCTCATCTATGCCTTTCAAAGCATTCTTTAAATATGGCGGGTTTATTTCACCATCTCGGTAATGCAGTATTCCACGTCGACGGTTTAACTCGTGGTTTATATGCTCAGACATCATGGGGATTGCCCTGTAAAAAGAAATGAAAAAAAATCCATTATTTTCATTATTTACAATCAATTCGTTATAGCTTATTAACTCATTTCAATTTCCCTTATCTCCTACAGTTACTAGTAATTGGAATCACAGAATTTGGCGGACATGATTTTTTGACGTTGGTGTCGGATTACCGAAATTAAAGCTACGTTCAAACAAAATTTCCACAATATCCAACAAACGAATACCCAACAATCAACAGAATAGCTTCCCCGACACTTGGGGAATATCTGTAGCCTGATAATATATAAGTAAAAGAAAAGTGCTAACATGCTAAGATTTCCTAAATCATTGCGGGGCAAGTGACGAACAATTATATGTCCCACTTGTCCAAGAGCAGAGACTAGTTCATTATTGGGATACCGAAATTT

>R=61_126_0_0_0_CG_24_bps_326

AAAAAGTTCAATGATTGAAAATAAGTTCAATGCTTGTTACCGGAAATATTTTTCCTTCTGGTAATTCAATTACTTTCAAAAACGATGGAAATTGCAGCAATTTCTAATAAGATTTAAATTTCTAAAAAAAAATTTGATTAAAGAGCAAAAGTGGGCATTTATATAATTTTAATATTGATGTTCCTACAGTAATAGTTGATTTGAGATTAATCAGAGTAATTTCACTGAAAGTAATATTTGCTTCTTAAATTTGCTTACACATTTTGAAAGTTATTCAGTAAGATGAATAGTCTCACACATTTTGTATTTGCTTATTTATATATAAT

>R=63_138_0_0_0_CG_28_bps_484 ATAAATTGTTTATTTCTTAATTTCATATTCAGTTATTTATTTGTTGAAGTAATTTTCGTAACTTTTTACAATTTATCTGTTAAAGTGGAAAATTTTTGTATTCTTGAAAGTTTCTTACATAACTATTTGCAGGATTTATTGGCTTTACGATTACAAAGATTAGCACCTTTGTTAGCTCATACTGATAAAGCACTCTTTGTTAGCGCACATTAGTGTAAGACTCTTTGGGTACATTGCTAAACAATGAGCACATGTACAATTCTATGCAGTGATAACATCACATTCATACAGAAAAAACTCATGATTTAACATGGTATATGCAAATTAATACGTGCTCTACACATTCCTCATTCTTTGTCACGAATTTTTTCGCGTTTATTCGCGACATCTGTCGACAAAAAATTTTCTACACGGATTTATATGTATTTCAATTCAGTAATTAAAATGTTTTCATTTCAGCAGGGTTTAAATTTTTTCATTTATA

>R=66_80_0_0_0_CG_26_bps_322 TATTATGAGTGGTTTTAATTGAAATTCGTAAATTTCTCAAGTAAATGATTTGTTACATAAAATTTAAAAAATAAATATTTGACACCCAAATTGATCAACTCATCAGTTCAAAAAGAAGTCCTTTTCGACAAGTGGGTTAAATAAACCATCAATCCTATATTCCGGAATGCAGCACTGAGATCAAAATTTACTAATATCATTATTCATTTAGTAACATTTCACATAATTCATAAGTCGTCTCACAGCATATTTAGAAAAAAGAATATCAGAAATTATGTTAATACCGATTTATGAAATGAGAAGGAACAATGTGAAAAGAAAT

>R=68_254_0_0_0_CG_41_bps_321

TGTCGCTGAAATCACGACGAGAAGCAGTGAATCAATAATTGCTTTGATACTGTTGGTATAATGTATTGTATGTATAGATATACTGTGCAATGTAAACTAATATTGTCCATCCTGTTTTTTATCTCCTATAATAATAATTCTTGTGACCCCAGAAGTGACCTCAGAATTGACCTCGGAACTGACCTCAGAACTATCCCAGGATCCGCCCAATTCGAGGAAGAAGAAGGTCCTGAGCGCCAAATCATTTTCGAAAGACGTGTTCCAACAGGTCGGTTGGAAAGACGATAACGGGCGTCATTTGGTCTTTGGTCCCCCTTTTGT

>R=75_82_0_0_0_CG_30_bps_458

TTTCGTTTGTCAACTCACAGTTCCATTGCAAAGTTCCGTTGAAGACAATAGGCATTTTAATTATGTTATTTGATGTACATTAGTACTAATGCATAAATTCATATCAGCAAAGATTACTTTTAACGGTCGTTTTCAGTGCTCTTGTGAGATTTGGTTTATAGAATTTTGAGAATCATAAAAAGTGCTTCGTTAGGTATTATTCACTATTCCATGGCAAAATGTGAACAAATATTGCATTTTTTGAACAAAAAAAAAATGTGTAAAGGAAATAGAGGAAGGAAATTAGAAATTCTGAATAGCACAATATACTGCTAAATGTTCCAAAACTCTGGAATAGAAATTGAAAATTCCCAAAATTTCTACCTACTTTGGAAAAACCGAACACGGGAATATTTATATCAGCAAGATTACTTGCATCAGCATATATCCGATTTTCAATGTTATGAATGAATTTCAGA

>R=84_72_0_0_0_CG_27_bps_525

AATTTATTAGGTTCTGTAAATATTTTTGCCGATTTTCTACAACAAAATATTACCAGATTTATCCGTAACACTAAAAAATCTTTCACGATAATAAAATTCGGATCCATTTGAAAAGAAATCACTACCATCTCTAGATGTAATTGCTCTTCCAAATTTCCGCTTTCATTCTTCGATCTGTTGCTTATAGATCAAAGATCTTTTAACCGAGGGATTCAGAATTTGAATGACAAGAAGGCAATATAATGCATATTTGAGAGTTCAAATGTATTATATTTGCGATTAGAACAACACACAAAGGAACATGTACGTGTGCTTTCAAAGGAACGTGTGACAAGTCGACAATGTATAATGCATATTTGAGAAGAGCCAAAAGTATAATATATTCACACGTATAATATATTCATATATAATATATTCAGTATAATATATTCACACGTATAATATATTCATATATAATATATTCAGTATAATATATTCACACGTATAATATATTCATATATAATGTATTCAGTATAATATATTCAT

>R=89_264_0_0_0_CG_30_bps_374

CAACATAATTTTGGTTGCTTTTATTTCTGACACAAATGCAAATTTACTTAATTCAACGAAAGACTTCCAGCGACACGAAAAATTCTGTTAATCCCAATTTCTACAGAAAAACTGTTTGTGACAATTCAGTGAAAGGCTTCCAGTGACACGAAATATTCTGTAAATCCCAATTTCTATAGAAAAACTGTTTGTGACAGTATCATTTGCTTTTTTTCCATAATCAGTATGGAATAATTTTGTTTTACAATGTATTGCTATTCAATACAAATTGGAAAACGGATTAATTTCTATTTCCCGTTGTTTTTCGGAAAAGTTGTATGGAATCTGTTTATCGCAAGTTGCCTACTCCTTAATGTAAAATATTTTCTGGAAAA

>R=99_70_0_0_0_CG_30_bps_361

TCATTAAGCATTTATCACAGATTATTCTTAATATGGCTCTCATATTCGGCATATTTCCTTCCCTATGTTAAAAGATCATCGATTTAGCAGCAGCAGATCGAAGAATGAAAGCGAGAATTTGTAAGAACAATTGCATCTCGAGATGGTAGTGATTTCTTTTCAATCGGATCCGAATTTTATTATCGTGAAAGATTTTTTAGTGTTACGGATAAATCTGATAATATTTTGTTGTAGAAAATCGGCAAAAATATTCACAAAACCTAATAAAAAAAAAGTAAACATGAATCACATTTCTAATTTGACATAATTTTGGTATCGCAATTCTACATTCTCTGAAATTATGGCTTTGGTCCATACAAAC

>R=102_94_0_0_0_CG_31_bps_423

GAGTCTTAGTTAAACGCTCAATGACATTTTCCAAAGGATTGGAAATGAAAAATGTGCAGGAAGTGGAAATTTAGAAAATTCCGCATATTATTAACAGGCTCTTATTTCTTTTTACAGTCAATAAATTTTCGAACTGTGAATGAAGCGACTATGTTCCAGCTTGTTATAACTTTAAATTTACGCTTTATAACCCATCAGGTGGATTACCATCTGATCGGTTAATATCCTCAGAAATCGTTTCAACGAAAACTTAGCACACATATTTTCTGGAAACATTTGGTGTTTCCAAAATACCGCAGATCCATTTTATGATATTCAGAAAAATAATAACTGCAAAAGGATTCAAAAAATTAATATTTAAATCAGCTTTTATTATTGAATTCGAGCTCTCACGTTATATGGCATAATAATGATTGAATGGAA

> R=134_52_0_0_0_CG_29_bps_713

AAGTTGACCTTTACTAACTTTAGTCATTCTTTCATCTTTTTCCAATTATCATGAATCATTTTTTCCGCGCAAAAAATCAAAATGATGTCAGGAAGCGCCAAAAACGATAAAAATCTTCTGAGAAATGACTTTTCACACCTCTTTTTACTACAGCATTTATCTTGAAGATACTGTCTTTTTTGTTGCAATTTTTTGCTCACAGTTTGGTTCTGTTTTAATTCTTTATCATAATTAATTAAATTAAATGAGATCGATTTGTATGTATTATCTCCATCTGTTAAAATATCAACCTGATAATGAAAACCGATGCATAAGTCAAGTTGAAAATAGTAATGTGAGTTTTTAAACGACACAATTTCTACTAATTCGCCAACCCACAATTAATTAAAGTGAATATTAACTTATTAGTAGGACGAAAGAAGATATAAGTAGATCGGCCAATTCATTCGATTCAGTCGTGCTCTTTTATCAATCTGTTTTTCGCATTTCCTCTTTATCAATGTTTAAATGATAGAAAGACCTTAAAATTCAACAGTTTATTTTAACGTTGCTGTCATCACGACTTAACTGATAACTTTGCATGGAAGTAATTTAGCATCTATTAATTAATGACTTTAATCTTTGCATGTCTTTCTCTGTTTGGTAGAAACTGCACCAAAAGCATTAATTAGATATATGATAAAAAGTTCAAAGAGATCAATTACCATAAAAGA

>R=140_226_0_0_0_CG_30_bps_634

CTTACAACTTATAATAGATTATCGTCTGGATACCAAATACAGTAAAGATAGGTTCAAATAATTCCAATTACAATAGAATGACTTACAGGATAACTTCCAGAGAAAAGTGAGATTTAATAATGATTAGTTGAAAGGGATAGAAAAATGAAGAGCGTTTGTGTGAACCTGAGTGTCGACAGATGGGATTTTTGCAACAAAAGTAAAGACTGTTAGGATTTCTAACGTTTATATAAGGAGTGTGTAAGGAGAAACAATGATAACAAATTAACGTATACTCACAGCTACCAGAGAAATCTCCATATCTATATTAATTTATATTAAGTGGCTTCAATCACAATATACTTTGTTTGGAGAAAGGAAAGAAATACAATAATTAACAGAAATCTAGGGATAAATATGTTGGATATAAACATGTTTATAAGATCAGATATCTCTACAGTATGCCCATCAATGTGCATAAAATTAGTTGTCATCTAGATATGTGCAAACCTCAATTTCAAATAAAGCTCATTTAGGCGATTTAAAGATTTGATAGCTGACTCATGACCTTAGGCATGAGAGAGAAAAAACAAATGTAAATGGAAACTATTTGAATGAATAATTTCATAATACAATTATTTCGGTTTTTACGACA

>R=143_60_0_0_0_CG_35_bps_315

AGCATCATTCATTAAACCTATCAAATAACTTTTTGAGTAACTTATATTACGGGATGTTATTCACTATTATCACTTAAATCGCGAGGGGAAGCAGTGAATAATTACTTTGATACTGCTGTTATAATATATTGTATATGTAGATAAACTATGCAATGTAAGTTAATATTGTGGTGCGCCCATTGTCCCAGATCTCACCTCAGAGCTGTGTCAGGATCTCTGAATTCGAGGATGAAGAGAATCCTGTGCACCAAAATCATTTGGAAAAATGTATTTCGTCGAAGTCGATTAAATATACGATATCAGGGTCACTTGGTC

>R=147_207_0_0_0_CG_30_bps_511

CTAATCCTTGTAATCGTGTAATAATATGCATATAAAGCCTGCAGATAGCAGAAGGACATTCTTCCTTTCTTTTATTCTCCAAATTGATGTCTTCGAAATTCATCTTGTGGCTTATGTTTATGTTTTTGGAATTAGCTTTATGCATAATTTGTGTTTTGTAATTCTTAATGATTATGCTATGCTAATATTGTTTTACTTATTTGATAGATTCAATTATCCAAGTTTGAGATGAAAATAAAGAAAATCTTACAACTCTGTGTGACATTACACAAAATATTCCTAAGAATACAAAAATTTTCCACTTTAATTCACGTTCTGTCCCATTTTGTTGATATCAAAGTTGTACGTCTTTTCAAACTGATATCTGTCCAAATGTCTCAACAATAATTTTCTAAAACGAAGATAATGGAGAAACAGAAAAACAAGGGGAATCTCGACTTTCCTCATTTTTCACACTCAAATTTTTGACACGTTTGACGTGGCCGTACACGTAGGTCATCATTTCATCATT

>R=172_180_0_0_0_CG_29_bps_444

TAAGAAGGTGAGGGAGATTGTTTTTAGTCGTTGAAGAAGCAGTTAAATTTTAAAGGAATGACTTACAGAGTACCGGTATTGTTGTGTGTTCGAAACGTATAGCAGTTTATCCTTTATTATCGTCCGGGTACCAAATACAATAGAAATGAGTTCAAAATAATTCCAATTTCAATAAAATGACGTCCAGAATAATAACAATAGAACAATGAGAGAAATTTGTAAGTAGTAAAATAGTGAAATGGGAAAAAAATGAATGGTGTGATGTGTGAAGCTGAGTGCTGACCGATAAAAATCAATGAATCAATGTTAGAATTTCTTATGGTTTATATAAGGAATGTGTGAAGAGAAACATTCAAAACAAATTGACATATTCTCAGAGCTCCCAGAGAAATCTAAATATCTATATTAATTTATATTAAAAACAAGAAATTTAAATAACAATAT

>R=181_84_0_0_0_CG_32_bps_325

CAGAATAATACCAGTATAACAATGGGCGAAATTTGTAAGTAGTAACGTAATGAACTGGGAGAAATATGAAGAGTGTGTTGTGGGAGGCTGAGTGCCGACCGATAGAATTGAATGAATTGATGTTAGAATTTCTTATGGTTTATATAAGGAAATGTGTGAAGAGAAATTTTGTAACAATCATTAGAAATTGACAATAGTCTCACAGCTACAAAAAATTTACATACCAGGCATAAGATTGGTTTTATAGATAAATAGGAAATTACAGGAAATTATTTTGTGAGATGTACAGCTTCCTGAAGCAGTATGCAAGATTTGTGCATAAGAT

>R=183_64_0_0_0_CG_34_bps_409

TGTTATCAATTTATTGTCATCCATATGATTCAAAATTTACTAAAGCATTTAACTTTATTGGCATATTTTAAGTTTCATGGCCTATATAAAATTGAAAATGATCACAACACATTTGTGCACACTACATTTATCCACGCGATATTTCCGCACACTACATTTGTACATGCCACGTTTGCGAGCGCAACATTTGCGTACACGGTTCTTGCTCACGCGATGTTTGCGTGCACGACATTTGCGTAAAAATATAACAAACATGACATTTCCGCAACCATCAAAAATTGTCACATTGGAAAGTATCAATGGGGAAAAATCCAAAAAATGTACATTATGGAAAATTCCAGGAAATATACTCTATTGAAAATTCCAGAAAATGTCTGCTATGGGAAACTCCAGAGAATATCTCTTCTAC

>R=198_162_0_0_0_CG_35_bps_524

AAAAATCCGAAACAGTTCAGTTTTTAAAAATCAAGCAAACTATAATCCTCTTCTCATATCCGCCACCACTGAACAACAAACTACAATATCTTCCTTTACTCAAAATATTTGCTGCAACATATCTCCAGCTCAACGAAGGATGAATTCTTCGTTTTTAAACAATTACGCAATCTATCAACAAAACGACAGTCAACTACTATCGAAGGGAATGACAAAGTATTTAATAGTTGGTCATTTTGCTTGCTTATTGTATAGCTCATTCATCACTGCTTATCAGCTCACCGTGTAATATGCATAATGCGTATTTTTTATGTTCAAGAACATGACATCCGGCAAAAATTTGTATCGCTGAAAAGCGATGTCAACAACAGATTAGCTTCCCCGACCAACTCTATTACCTGACTTAAATCACTGATCCAACTATATTTGGCCCTGTTTCGGTTTTATCCTCCAGTAATTTATTTCAATCTTTTCCTCGAGATTGAAACAACGATCATGTCCATTCGTATTAATTTCCACTTAAT

>R=207_132_0_0_0_CG_28_bps_578

GTTGAATATATCAAGTATCGAATATCGAATATTGAATAAGTTTGACGATTACATTGAAATAATATTGTGGTATATTTTGTGTCGAAGTATTGAATATATCATATATCGAATATCGGATACTGAATAACTTTGACAATTATATTTATTGTGGCATATTTTGCATCGAATAAGTATTGAATATATCAAAATCGAATACCGAATATTGAATAAGTCTGGCGATTAATCGAATAATATTATGGCACGTTTTGAGCTGAATAAGTATTGAATATATCAAATATTACATATCGGATATTGGATAAGCTTGATGATTACATTAATTACTCCATGAAGAGAAATCATGAAAAGTGCTCATGTCACATTGTGACAACCTTACTATAATGATTAGCTAACAAATCGCTTAACATTTACATAAGGTGAAAGAAATCAATTATCTGGTTCATTTTTCGAAATAGTATCCATTGATGGTGGTAAAGAGAATTGAAACGGAATAGATATAATTTGGAATTTCATCAAAACATTTATTTAAAAATCTGATAGGCTTACTTAGACTACTAGCAATGTATTACTAGCAAGCCTTG

>R=233_160_0_0_0_CG_34_bps_335

TTTTGTTGAGATTGAATTATCAGTGGATGGGTGAGGATCTGTTGATCCTTTTGATGTAGCAGAATATCAGGAATACAGGTAGTCGTTACTTTAATCCCGAGGAATAGATTGAAATGAATTTTGAGGGAGTAAAACAATAACGGAGCAGAATAACAAATTGGATCGGTGATTTAAAATTCCCTTGCAGAGATGGATGCGTATCTCTTTTTTAGTGAGTCAACAATGAATCCTTTTATATTGAAAACAAATGAGTGTGGATTACAATATGAGAATAGCTCCCTATAAGGTTTTTACAAAGCTGACAGTAATTTCATAGATGCAAGAGAAATTTGCAT

>R=243_198_0_0_0_CG_31_bps_451

CGAAATAAAAATTTGGGAATCTGTTTATAAAAGCAAAAAAGAAAACTTTCATTAAATAAAACTTTCCCTGAATCGGAAATAATGACAATCGATGGCACAGCTTATCTTAATACGCTTCTATTTCTGATACAAATGCAAACTTATGTAATTCAATGAAAGGCTTCCAGTGACACGAAATATTCTGTTAATCCCAATTTCTGTAGAAAAACTCTTTGTGACAGTATTATTCACTTTTTTGCATAATCTGTATGAAATACTTTCTGTTTTACAATGCATTGTTATTCAATGCAAAAGGGAAAACTGAGATTTATTTCGATTTTTTTTGAGTCTTTCTGATATTTGACTGATTCGATCTTTTCACTGTAAAAGGTCCATATTTCCCTTCAGAATTCAGTCTACCGTCACAAACTGCTTTCTTCCTCACCAAATTCGTTTCCACAATTTCTTCTCC

>R=274_72_0_0_0_CG_30_bps_521

GTTTTATTTTAGTACTTTGAATTAGCTTAAGTTTAGAGATAATTGAAGACAAATTGGAGCTGAAAATAAGGAGACGGTCGATCGTCGGAGCCTGTAAAGCAAAAAGTGATTTCAAACTTCGCATATTGTGAGTGTGGTTTACAAAACCCATATATTACATAAAAAATTAATTCTCAAACAAAGGAATTCTAGAACAAAAGGAATGCCACTGGATATAAGCACCACTGAATATCAGATTAGATACTCTTACAATATACCCATCAATTTGCATAAGCTGGATTGTCATACCAAGATGCACACTAAGGACCAAAGATTACAATAACTTTGTTATTAAGCTAGTGTCCTTTTCCCATAGTGTTACGAGAAATGCAAAAATGACCCATCATTTAATACGTTAAAAATTCAATTGTAATATTTGTATAATAAACCATTATCCAAAAGTGATCTAAATAAGGAATATTTAAAATGCAAATTTGTGATACTTTGATTGTTTTAACATATTGTAAATTTTCTCGGGTTTT

>R=275_84_0_0_0_CG_29_bps_554

TTACCAGATTTATCCGTAACACAAAAAATCTTTCACGATAATAAAATTCGGATCCGATTGAAAAGAAATCACTACCATCTCGAGATGCAATTGTTCTTACAAATTCTCACTTTCATTCTTCGATCTGCTGCTGCTAAATCGATGATCTTTTAACATAGGGAAGGGAATATGCCGAATATGAGAGCCATATTAAGAATAATCTGAGATAAATGCTTAATGACATTTTAAAAATAACATAATAAATTATTGAAGAAGTCAAATTTACGTATATTTCCGATTTCATCAGAAATTTATGCTGGATGATGCACTGTAGAAGCCTTTAATCATTTATAATATCCTGAGGGAGTCACTGTGATTGACGAGGAAAATTAATCTTTGACTAATGTAACAGTCATCAAAGATTACACTGCGATTAATGTTTGATAACACTAATTTGGATAGTATTTGATTTTCAATGCTGATTCTATTGTTACATGTCCACTTTGATTAATAAATACAAATACCATATGTTTAACATATAATTTTCTTCTGTTTTTCAACATTAGCAATTTCGT

>R=295_110_0_0_0_CG_36_bps_308

AATTAGAATTCATATTATCAAATAAATATTTTGATACCTAAATTGATAAATCATTTACTCATCACATGCATTTGTCGGAATATTTTAAAAAAGTTAAGCGCAAATGTCCAAATCCTGTAAAGAACTTCTAATCTACCAGTTAAGCCAATCTGTCTGGAAAGTTAAACCACTCCCACTGTAAAAGGAGCTAGGATCAATCATCCGGACGATCAGTTACTAAATCCAAAAAGCTCGAGCAGTCACTTGATCCGATACCTCCCTAAATCACTCAGTTGGACAGCTCACGCAAACACTCAAGACGACATACA

>R=333_56_0_0_0_CG_32_bps_419

ATCCCTTTGTTAACGAAAAACTTCGAAATTACTATAAGTAGTTATCCCTACGATTTCGTCACGTTGTTACGAATAAAGGCGAATTTCCGTGTCATAAATCATAAATGAATATGTAATGATGTACTGTACCATATTGGATCATGATTGAGGTTAAATATCGATCGTATGAATAGGATAATATCATTGCATAGAATAATTGTATACGTACGTACTCTTTGTTTAGCAATCTATCTAAAGATCCCTTCATTAATGTATGCTAACAAAGAATGCTTTATCAATATGAGTTAACAAAGGGGCTAATCCTTGTAATCCTTGAAGGATGTAATGCTGATAGTAGAACAGGACATTCTCCTCTGTCTTCTATTCTTTTGATTGGTATCTTCGAAATTTATCGTCTGGCAATGTTTTTGGAATTCTTT

>R=339_120_0_0_0_CG_33_bps_940

ACCAAACAAAATAGCTTGACCTGGTCTCCAATGATCCATCGCAAAGGATAATCGCTCCTAAAGTTTGAATTTGCATCATTACGTCATCAAACCTTCGTCTCTCATAAGTTCTCTCTGATTTTTAGCAAATTATTATTCCTGACAGAAACAAAGAATGAAACAAGCCCGAAAATCGGTGAAATTCATCTTAGATACGAGTTCCAAAGCCTTCTTCAAATATGGCGGTGTTATTTTACCGTCCCGGTAATATAATATTCTATATCGACGGTTCAACTCATAGTTTGCACATTCAGACATCTCGGAGATTTTCCTGTAAGATGATATGAAAGAATAATCCATTATTATTATTACCAATCAATTCATTATAACTAATTAACTCATTTCAATTTCCCTTATCTCCTACAGTTATTAGTAATTGAAATCATCAAATTTGGCGGATATGATTTTTCCACCTTAGGGAATATATGTAGTGTGATAATATATAAGTGAAAGAAGAGTGCTCACGCCATATTTTTTAAATCACCGGTAGGTAGGTAACCAGCAATTTGATTTCCCACTTCTCCAAAAACACGGTTTACTTTATCGTTGGGACACCGACATTTAATTTCAATCTTTCTTTTTTTAGGAGCACTGGGTCCTTCCTCATCTGTTTCTCCTGAAATTTCAATTAATTCATCACTAAAGTTCTTGTTCTTTACATATTTGAACAGGAAGTATAAATTCATTCAAATTTCCTAATATGCACAGTGTACTACTTCAAAATAGTTCAGTTAAATTTTAAATTTTGAAAATTCAAAAAGTGTACCTGTCGCTGATGCAATGTTGGATAATATTACGACGAACGTACGGAAAGCAATTTAATCGAAGTGCATTGCTCTCAGCAGCTGAAAATTAAAATTTTACTCAGTGATTTGATTAATCTTACGAATTTGTCAAATTT

>R=349_78_0_0_0_CG_28_bps_497

AGAATTTAACTAATGATATTGTCAAGGCAAAGAACACATTCTCGATCTTACTTCAGACTACTAAAATTGTCTTTCGGATAGCAGAGATCACGTTTTGTATACAAGTACCGATTTTAAATGAAACAAAAGATTTAGTAATAGATATAAGCTCTCAAATATGTATTATATTGTCTTCTAGTATGTGCATATCGTTCATTTCCTAATTCCTCAGTATGTTTTTCCTTTCCTATGTTAAAAGATCTTTGATCTGCAAGCAACAGATTGAGGAATAAAAGCAGAAATTTGGAAGAGGAATTGTATCTAGAGATAGGTAATGGTTTTTCTTTTCAAATGGATCCGAATTTTAATATCCTAAAAGTTTTTTATGATATTGAGGATAAATCTGGTAATATTTTATTGTGAAAAATCGGCAAAAATATTTACAACACTTAATAAATTGATGAATCACGTTCCTAATTTGATATTATTTCGTATCGCAAATCTCTTTGGCTATTCAA

> R=352_54_0_0_0_CG_33_bps_624

CTAATTTGCAATATAATGTCAAATCATGGTATTTCATAAATATGACTCATCTCGATTTGATAGTTAAAGATGTGTTTGCATATTTGTATCAGAAGTAAAACAAAATAAAATCTCATGAAAATGGTTTGAAAAATGAGCTGAAAAGTCGAAATGAAGTAGAAATTTGTGCTAGTAATCCCCTCGTATTGAAACTTTGCGACCACAGCTTAAAATAGCAATAAATGAAATTAAATCGTTGTAAGTCAGAGTTTGGAAGTTAATAATTGATTTTTTGGCCTTGTTCAGATCATTGATTTTGGAAAATTTAAACTGAAACACTGCTTAATAAAAATTGAAGTAACTCTGAAGAACACTTTGAGAAAAAGCAACTCTCTAGAACTGTGTAGAAAGAATAGAAATGATCGCCATCGAAGCAAGGGAATAGGAAAAAAACGATTTGATGAGGTCGTTCAGGGTCATGAATGAATCGCCTGTCTACAATGGATGGCCTTGCATAACTTATTAACTCATTTCCTCTATGAAGTCTTCCGATTTATGGTATTTTGTTCCCAAACTATGTCCACCACGTCATTTTGTTTCAGCATCGTTCTCCCTCCCACAATTATGCTTAATCATTTGTCACCA

>R=367_134_0_0_0_CG_36_bps_440

AATTAGAATTCATATTATCAAATAAATATTTTGATACCTAAATTGATAAATCATTTACTCATCACATGCATTTGTCGGAATATTTTAAAAAAGTTAAGTGCAAATGTCGAAATTTTGTAAAGAACTTCAAATCTACCAGTTAAGCCAATCTGTCTGGAAAGTTAAACCACTCCCACTGTAAAAGGAGCTAGGATCAATCATCCGGACGATCAGTTACTAAATCCAAAAAGCTCGAGCAGTCACTTGATCCGATAGCTCGCTCAATCACTCAGTTGGGCAGCTTGCGCAAGCACTCAAGACGACATACAGTCGGGCACTCAATTCTGCAGTGGTCAGTTGCAGAAAAGCAAGACAGTTGAACAGAAGAAGCAAAAAGACCTTATTAGTAGCATTTTGTGCGAAAAAATAAAATAAAAAATTTTGGTTTGTCGGGTTTGATT

>R=389_160_0_0_0_CG_33_bps_363

TTTTTACATATATTGAAAACGAACTAATATGCAAATTTCTCTTGCATCTATGAAATTACTGTCAGCTTTGTAAAAACCTTATAGGGAGCTATTCTCATATTGTAATCCACACTCATTTGTTTTCAATATAAAAGGATTCATTGTTGACTCACTAAAAAAGAGATACGCATCCATCTCTGCAAGGGAATTTTAAATCACCGATCCAATTTGTTATTCTGCTCCGTTATTGTTTTACTCCCTCAAAATTCATTTCAATCTATTCCTCGGGATTAAAGTAACGACTACCTGTATTCCTGATATTCTGCTACATCAAAAGGATCAACAGATCCTCACCCATCCACTGATAATTCAATCTCAACAAAA

>R=396_148_0_0_0_CG_37_bps_369

ACGAAATACATCAATCTAAAGCTCAACGTGAAAATTCTAGCGATTCCTTTCCTTCTAAACATAAACTACGAGAATATAATCAACGATGCCACGGAGTTAGAGGTTATTTTCAGAAGTTTATGTTTAGCAAAAGAAGTTTCAACGTGTTTGTTCACATGAAAACATCTGCGATAGCAAAGTGGCAAATCAAAATTCAATACTCACTGCAGCATTTTTCTAACCTCAGTAGATAGCTGCTTGTCGTTTATCGCAGTGGATGTTTGGCAGTTACTGATATGACAATGAACGGAGTGACAGAATCCGAGCAATTAGCGTCCTTACAGTCCGATGAATTTAAATGGTGTTGACTGTTGGGAAATTTGTTTTTCT

>R=424_74_0_0_0_CG_31_bps_554

TAATGTTCTTGTTTTAGTGCACTATCTGTCGGAAAACCCGAGAAAATTTGCAATGCGTTAAAACAATCAAAGTATCACAAATTTGCATCCTAAATATTACCTTATTTAGATCACTTTTGGATAATGGTTTATTATACAAATATTACAATTGAATTTTTAACGTATTAAATGATGGGTCATTTATGCATTTCTCGTAACACTATGGGAAAAGGACACTAGCTTAATAACAAAGTTATTGCAATCTTTGGTCCTTAGTGTACATCTTGGTATGACAATCCAGCTTATGCAAATTGATGGGTATATTGTAAGAGTATCTAATCTGATATTCAGTGGTGCTTATATCCAGTGGCACTCCTTTTGTTCTAGAATTCCTTTGTTTGAGAATTAATTTTTTATGTAATATATGGGTTTTGTAAACCACACCCACAATATACAAAGTTTGAAATCACTTTTTGCTTTACAGGCTCCGACGATCGACCGTATCCTTATTTCCAGCTCCAATTTGTCTTCAATTATCTCTAAACTAAAGCTAATTCAAAGTACTAAAACAAAAC

>R=426_396_0_0_0_CG_34_bps_997

TTTCACTGAATGGTTCAAAAGAGTAGTCCTAAGTAATAGTAAGAAATCAAAATTATGCTAAGAATATAGCAAAGAAGTTTCATTTAAATATTTCAAAAATTTGAAGTTTAAGCATGAATTATTAACCTATTTGAATGTAACAATAAAGATTTTCGATTAAAAAAGTTTTCCACTAGCGAGATCAGAGCAAGGAGATCAATTGTTGTTTCAAGAGTGATGAAATTATCGAGGAAAAACAAGGCAGTTTATTTTGTGCTGTTGTAACTCCGCATTGCTGATTTTAACCTTTATTGGTGTTATATGATATGTACAATATCGTACTGAAACAATGTCACAAAGCTCCAAATGAAATTTCTTTGTCTGAGAACTAATCTTAATTGCAACAATGCTCAATGTTGCAAACAGAGCCTTTTGTTGCAATCGGGTTTCGACTGTTTTTCTTCATTCAAAGGATAATCTGATGTAATGTTTGTTATAAATCACTCTCTCCAAATATATATACACTTAAGGCTATAACCAGATCATTTCCTTTTCAAACACTCAATCATGGACTGCACTATGAATTGTATCCTCTACTCCAATAAATATTTTTACTACTATCCTCTGTGTTATTACAATCTACTATTCTGCGAATTAATAACTTCTTCCTGAACCGAACGAAGAATACCTTCTCAGAATATCCGTCTGTGCCTTCCGAATCAAACCCGACAAACCAAAAGCTTTTATTTTAATTTTTCGCACAAAATGATACTAAAAAGGTCTTTTTGCTTATTCTGTTCAACTGTCTTGCTTTTCTGCAACTGACCACTGCAGAATTGAGTGCCCGACTGTATGTCGTCTTGAGTGCTTGCGCAAGCTGCCCAACTGAGTGATTGAGCGAGCTATCGGATCAAGTGACTGCTCGAGCTTTTTGGATTTAGTAACTGATCGTCCGGATGATTGATCCTAGCTCCTTTTACAGTGGGAGTGGTTTAACTTTCCAGACAGATTGGCTTAA

>R=459_62_0_0_0_CG_31_bps_813

CTAATTTGCAACATAATGTCAAATCATGGTATTTCATAAATATGACTCATCTCGATTTGATAGTTAAAGATGTGTTTGCATATTTGTATCAGAAGTAAAACAAAATAAAATCTCATGAAAATGGTTTGAAAAATGAGCTGAAAAGTCGAAATGAAGTAGAAATTTGTGCTAGTAATCCCCTCGTACTGAAACTTTGCGACCACAGCTTAAAATAGCAATAAATGAAATTAAATCGTTGTAAGTCAGAGTTTGGAAGTTAATAATTGATTTTTTGGCCTTGTTCAGATCATTGATTTTGGAAAATTTAAACAGAAACACTGCTTAATAAAAATTGAAGTAACTCTGAAGAACACTTTGAGAAAAAGCAACTCTCTAGAACTGTGTAGAAAGAATAGAAATGATCGCCATCGAAGCAAGGGAATAGGAAAAAAACGATTTGATGAGGTCGTTCAGGGTCATGAATGAATCGCCTGTCTACAATGGATGGCCTTGCATAACTTATTAACTCATTTCCTCTATGAAGTCTTCCGATTTATGGTATTTTGTTCCCAAACTATGTCCACCACGTCATTTTGTTTCAGCATCGTTCTCCCTCCCACAATTATGCATAATCATTTGTCACCAACTTAATCGCTTCCTACTACCAGGACATATATCTCAATCTCAAATCGTTATTATATGAACATTATTATATGAACAGGTATTATATGAACATTATCGTTATTATATGAACAGGTAACATGAAAATAAATTATGGTTACACGAACTAATTCATTCAAAGAATTTTGATTCACAAATAGTTTTTGCAACTTT

>R=476_260_0_0_0_CG_29_bps_328

AGCCGAGAAAATGCTATGAGTTGTTTTAATTAGAATTCAGAATATTTCTCATACAAATGGTTTGTTACAGAAAATCAATAAAATAAATATTTCGATACCTAAAGTGATGAATCATTCATCCAAATCCAACCCTCCAAATTTTTAACCAATCTTAACTGCAATGTTTAAATGTTGTGGACAAAACCCTTTGTTGTGAATCTTTTTTTTTTTCAATCCATTAATCAAGCTTACATGCAATTGTCGGAATGTTGCAAAGAACTTATGTACAAATGTCAAAAACCTCTAGTCTGCCAGTTATCTAGAATGTTAAACCATTTCCACTATAAAA

>R=528_66_0_0_0_CG_31_bps_802

TGTGCAGCAAATCATAATCTTTCTTGATAAAGTTTTCTTGGTTATTCATCATGTAGAATTACAGTATTCAAGAATAAATTTACTTCATTATAACAGTTATGAAGTTGAGGAAAGTAAATTAGAAGTAAACTGAGTGCTGGCCAAAATTAACAAAAACCACTGTGTATACCCGTCAAAGTTCGAGATATCGTCCTCAAGTTCATGTCAAATTGTTATAGGATATGATGTGGGAACGGCATTTTGATTTAAGATTCAATGATACGAACAGAATTAACTTACTAATTATCCAGTTTTTAATGACTAAATGGGTTTAAAGAATATCACAAACATTTATAATATACTGTACAGGCTGCGTGTGCTAAGTATTGAATGTAATGACCTCTAATCGGTTAATTAGCAGGAAAGTAATTGCTTCCTGACCAATTCATTATTAATCATTCAACAATTTCCTCCAATCAATTAGACAAAAACTTTTTTCTTAGCTTAAGGCCTCTGAATTAAAATTCATTCAGCATTACAAAAGATCACTAGACTTCACAAGTGAATCAATCACTGATTTGTATTCATTTGTAATTTTCAAAACTTCTATAGTTCCATGGAGAATAGCTCATATCCCCTACTCTCTTTGCATTCACTTTAAGCCAGATTCATTACATAAGGGAAATGATAGAAAAAGAGAACGTAAATTCCTGAAGAGCAGCATGGACATCGCATAAATCCATTCATAAATGTCACTATTCCAACAAGGAATACAAAAAATTAACTCAATATCTCAGTTGAAATCGGAGTAAATTAATGACGT

>R=571_144_0_0_0_CG_36_bps_445

TGTTGGACTGTCGAATTGCCGTTTTTCGCCGTTGAACCGTTGTCAAATTGAATACTTGGTTAAGCTGTCGCCTTGAGTGCTTTGCGCTAGCTCTCCGATTGAATGACTCAGCAAGCTGTCGGATCGAAAGACTACTCGAGCTTTTGAACTTGATAACTGACCACTAGGAGGATAACTGACCCTAGCCCCATTTATAGCATAACTACCACAGTAGAGATCCTTTATAAGATTTCGATATATGCGTATAAGTTCATTACAACATTTCAACATCAATTACACGTAAATTTGATTAACAGATTGGAAACAATTTACATCAAAGGGTTAAAAAATTGGAACAGATGGATTTGAATGAGTGACTCATCACTTTAAGTATCAAGATATTTATTTCATTAATTATGTATAACGAGTCTACTTATTTGGGAAGTATTCTGAATTCCAATTCATG

>R=635_258_0_0_0_CG_30_bps_392

AGAATTCAAATATAATGCTTTTCCAGAAAATATATGACATTGAGGAGTAGGCAACCTGCGATAAACAGATTCCATACAACTTTTCCGAAAAACAACGGGAAATAGAAATTAATCCGTTTTCCAATTTGTATTGAATAGCAATACATTGTAAAACAAAAATATTCCATACTGATTATGGAAAAAAAGCAAATGATACTGTCACAAACAGTTTTTCTATAGAAATTGGGATAAAAAGAAAATTTCGTGTCACTGGAAGCCTTTCACTGAATTGTCACAAACAGTTTTTCTGTAGAAATTAGGATTAACAGAATATTTCGTGTCACTGGAAGTCTTTCGTTGAATTAAGTAAATTTGCATTTGTGTCAGAAATAAAAGCAACCAAAATTATGTTG

>R=658_112_0_0_0_CG_31_bps_376

ACGTGCTAAGAGAAATTGTTTTGTTAAGATTAAATTATCAATGAATGGGTGAGGATCTGTTGATCCTATTGATGTAGCTGAATACCAGGAATACAGATAGTATTTATTAACTAAATAGAAATTAACGCTGATGGGACACAATCATTACTTTAATTCTGATGAATAGATTGAAATGAATTATTGGAGAATAAAACAGAAATTGAGCAAAATATGGTTGGTTGGTGGTTTAAGTGAAGAAGTGGAGATTCCTTCTTTGCACGGATGGATGTATATCTTTCTTAAGTCTATAGTAAGCTCTCATCTCAAATCTTAGTTAATTTATATTGAGAACAAGTGGGTGCAGATTATAATATGAGATGCATATAAGGTCTTTACA

>R=679_120_0_0_0_CG_32_bps_964

CGAAATCCATTTATGATGGTGAAAAAATTTGACAAATTCGTAAGATTATTCGAATCACTGAGTAAATTTTTAATTTACAGCTACTGATAGCAATGCACTTCGAATATTTTGCTATCCTTACGTTCGTCGTAATATCATCCAACATTGCATCAGCGACAGGTACACTTTTTGAATTTTCAAAATTTAAAATTTAACTGAACTATTTTGAAGTAGTACACTGTGCATATTAGGAAATTTGAATGAATTTATACTTCCTGTTCAAATATGTAAAGAACAAGAACTTTAGTGATGAATTAATTGAAATTTCAGGAGAAACAGATGAGGAAGGACCCAGTGCTCCTAAAAAAAGAAAGATTGAAATTAAATGTCGGTGTCCCAACGATAAAGTAAACCGTGTTTTTGGAGAAGTGGGAAATCAAATTGCTGGTTACCTACCTACCGGTGATTTAAAAAATATGGCGTGAGCACTCTTCTTTCACTTATATATTATCACACTACATATATTCCCTAAGGTGGAAAAATCATATCCGCCAAATTTGATGATTTCAATTACTAATAACTGTAGGAGATAAGGGAAATTGAAATGAGTTAATTAGTTATAATGAATTGATTGGTAATAATAATAATGGATTATTCTTTCATATCATCTTACAGAAAAATCACCGAGATGTCTGAATGTGCAAACTATGAGTTGAACCATCGAAATAGAATATTACATTACCGGGACAATAAAATAACACCGCCATATTTGAAGAAAGCTTTGGAACTCATATCTAAGACGAATTTCACCGATACTAAGGCATGTTTCATTCTTTGTTTCCGTCAAGAATAATAATTTACTAAAAATAAGAAAGAACTTATGAGAGACGAAGATTCGATGACGTAATAATGCAAATTCAAACTTTAGCACCGAGAACCCTTCACAACGCATCACTGGACACCAGGTCAAGCGATTTTGTTTGGT

>R=683_590_0_0_0_CG_33_bps_354

ATTGTTCTTGCACAATATTACTAGTTAACAACAAAAAAGGGTATAAAAAATATATGTATATCACACGATAAATTGATAAGCAGTAATGAGTTATACAAAAGCAAGCAATATGATCAACTAACAAATTCCTTGTCGTTGCCTTCGATAATAATTTTACTGTCGATTCGTTGGTAGGTCGTTGAGTTGGGAATATGTTACGTAAAATATATTTAGCAAATAGAAAGAATATCGTAGTTTGTTGTCCAGTGGTGGTGAAGCTGGGAAGATGTCGTTGTAGTTCAGTCGTGGAGAATAAGTGTAGAGGATGTATCGTCTGCTCGATTGTAAAAACACTAAACCCTTTTGGATTTTTCC

>R=688_88_0_0_0_CG_29_bps_578

TTAAATTTTCTCTTTCCCTTTTAATTTCTCTCGAGAATGTTGTGAAAACGAAATTGCTAATGTTGAAAAACAGAATAAAATTATATGTTAAACATATGGTATTTGTATTTATTAATCAAAGTGGACATGTAACAATAGCATATGAATTGAAAATCAAATACTATCCAAATTAGTGTTATCAAACATCAATCGCAGAGTAATCTTTGATACCTGTCACATTAGTCAAAGATTAATTTCCCTCGTCAATCACAGTGACTCCCTCAGGATATTATAAATGATCAAAAGAGCCTACAATGCATCATCCAGCATAAATTTCTGATGAAATCGGAAATATACATAAATTTGACTTCTTCAATAATTTATTATGTTATTTTTAAAATGTCATTAAGCATTTATCTCAGATTATTCTTAATATGGCTCCCATATTCGGCATATTCCCTTCCCTATGTTAAAAGATCATCGATTTAGCAGCAGCAGATCGAAGAATGAAAGTGAGAATTTGTAAGAACAATTGCATCTCGAGATGGAAGTGATTTCTTTTCAATCGGATCCGAATTTTATTATCGTGAAAGATTTTT

>R=723_82_0_0_0_CG_32_bps_376

CAGAATAATACCAGTATAACAATGGGCGAAATTTGTAAGTAGTAACGTAATGAACTGGGAGAAATATGAAGAGTGTGTTGTGGGAGGCTGAGTGCCGACCGATAGAATTGAATGAATTGATGTTAGAATTTCTTATGGTTTATATAAGGAAATGTGTGAAGAGAAATTTTGTAACAATCATTAGAAATTGACAATAGTCTCACAGCTACAAAAAATTTACATACCAGGCATAAGATTGGTTTTATAGATAAATAGGAAATTACAGGAAATTATTTTGTGAGATATACAGCTTCCTGAAACAGTATGCAAGATTTATGCATAAGATAAGGCTTTTACAAATTGTAGAACGAGACAGGTAACATTTTTAGACGATTTG

>R=737_274_0_0_0_CG_30_bps_433

CAACATAATTTTGGTTGCTTTTATTTCTGACACAAATGCAAATTTACTTAATTCAACGAAAGACTTCCAGCGACACGAAAAATTCTGTTAATCCCAATTTCTACAGAAAAACTGTTTGTGACAATTCAGTGAAAGGCTTCCAGTGACACGAAATATTCTGTAAATCCCAATTTCTATAGAAAAACTGTTTGTGACAGTATCATTTGCTTTTTTTCCATAATCAGTATGGAATAATTTTGTTTTACAATGTATTGCTATTCAATACAAATTGGAAAACGGATTAATTTCTATTTCCCGTTGTTTTTCGGAAAAGTTGTATGGAATCTGTTTATCGCAGGTTGCCTACTCCTCAATGTCATATATTTTCTGGAAAAGCATTATATTTGAATTCTTAGCGTTATGCTGATTATCTTTTTTAATTCCGGACAAAATT

>R=745_116_0_0_0_CG_27_bps_513

AAAAAATTTTTGTGGTTTTTTTTGGATTTCCTTGCACAGATTTCTATTTAGCAGAAGTCGTAATTAACAGTAGCAATTTATATGGTTTCTGGTATTAAACTTATCTAGCTTTTTTACGGTGCTATAGTGCAGATATTCATAAATTTTTAGCTGTATTTGAATTCAATTTTTCTGCACCTCAACTTTTCCGTTTCTTGAAATACAGAAAAAGATTTACTAACGACTATAAAATTATTTATAGAACTGGCAGACTAGACTGTTTCAAAGCATTATATTGTTTGACCGGATAGATGGTTCAAAATGATTTTACAGAAACCCGATGCATTTCAGTTTATTTTTGACCAACATTATATATAAATAAGCAAATACAAAATGTATGAGAATATTCATCTATTCAAATTTAAGAAGCAAACAACATTTTCAGTAGAATTACGCTGATTAATCTCATGTCAACTATTACTGTAGGAAGATCAATATTTAAATTATATGTTCACTTTTGCTCTTTAATCAAAA

>R=847_80_0_0_0_CG_29_bps_393

AGCAGAATCCTGGAGTATATTCTGCCCGATATTAGCGTATCAATGTTCTATTTTCCGCATTTCTCGAGTGCAACAGAAGGAGAACTAATTCCGACAGACAATGATATATGAAGAGGGTTTTGAATATCCAGTTTTACTATCCAGCGATAGTAACGTATTTATCAATCTTTCATACAAATTAACTGTGTTCACTAATTATGTTATTTTAAAACGGTCGATTCAAATTTTGAAATAAATACAACTGATTTCCTTTATGATTGATCTAACAAAATTTGAATAATTAGAATTTCCTCAATGCTATTAACAAATACATTTGTTGAATATATGGATATAGAAATCTCATCGTCTTTTATATTTAAAATACAATGGATTCTTTTAAAAGTTATCATCGAA

>R=867_76_0_0_0_CG_25_bps_439

ATTACTCATTACTTTATTTTCGCATGTCATTTATTCATTTATTTGCGTTTTTTTTTTTTGCATGCCATTTATTTTTACTTTATTTTTGCATGCTGATTTATTTGTCCATATTTTTTTTGTGCATATTTTACTACTGACTTTATTTTTTCATGCGTTTTACTCACTCTATTTCTGCATATTTTATTATAAAATTCTTTTCTTTATGCTTTTACTCACTTTTCTTCCGCATGCATTATTAGTACAGCTTTATTTTTGCATGCCTTTTATTTATTTTATTTGTACATATCATTGCTTACTTTATTTTTGCATGTCGTTCAACTAACTTTATTCTTACATATTTCATTATGAACTTCATTTTTGAATGCTTTTTACTCAACTTTTCTCTGCATGTATTATTACCAACATTATTTTTGCATGCCTCTTACTCACCTTTTTCTTT

>R=923_464_0_0_0_CG_34_bps_310

ATATTACTAGTTAACAACAAGAAAGGTGTAAAATATATATGTATATATTACGCGGTGAATTGGTAAGCAGTGATAAATGAGTTATATAATAAGCAAGCGTTGTGATTAACTAACAAATTCCTTGTCGTTGCTTCGATGATAGTTTGATTATCGTTTTATTGATAGGTTACGTAATGGTTGAGATATGAGAATTCGTCCTTCGTTGAACTGGGGATATGTTGCAGGAAATAATTTTAGCGAAAAGGAGAGATACTGTAGTCTGTTGTTCAATGATGGTGGTGGATATGCGAAGAGGATGTTGCAGTTTGCT

>R=924_74_0_0_0_CG_29_bps_337

ACTATTCTTTCATAAAAAACAGTAAATAGAATACTGTGATGGATTCACTGAAAATGTGGAGGATCTAATTACATCATTATTTTTAATGTTCAGATGATGAACTTGTGATGAAAGACCAATGGACTTTAACATTGAAAGATTTTGGCTGGCATACCAATTTATGATATGACAAATGAAAAGTAGATAAAAACTTGGGGAATCACTACATAAGAGCAGCATTGATTGAAAATTGTGGAAAAAACAAAACAAAACAAAGATAGATTGTATTCAAAAACATCTAAAACGTTTTTTCCAAAACAAAAAAGAAAAGGAACCTGGTCAATATATGGTCAGATAC

>R=933_56_0_0_0_CG_32_bps_342

AAAAAATATAACGAATCATAAATGTATTGCATTTGAATTTATCAATGTTGAATTATAATTATCATAATTAGGATTGATTATCATTCAAATTCTTTTTCTGTTATATGTAGAATACTATCACTATGCATAGAATTGTATTTGTGCTTATTGTTTATACATAATGTATGCTAACAAAAAGCGCTAGATTAATAATGTAAGCTAACAAAGGGGGAAAACAACAGAGAGATTTCGGTGTTTTCGCACGTCCGCAGCATGAACCATACCAAATGTCAGCTAACCTCCATGATGACCATGGTGGAACTTACCAAAGCTCCTCCTCTATACATACGCTCAGAAGAAAAC

>R=940_292_0_0_0_CG_32_bps_368

GGATAACTGAAGGAGCTGGGATGACTGATCCTAGCTCCTTTTATAGTTGGAATGATTTGACATTCCAGACAGATTGATTCAACTACCAAACTAGAAGTTCTCAACGATGTTGCGACATTTGCGCATAATTTCTTTACAACATTCCGACAATCACACGTAGGCTTGATTAAATTCACAACAAAAGGCTTTATCTGCAACATTTAGACATTGCAATTAAGATTGGTTTAAAAATTGGAAGAGTCGCATTTGGATAAATGATTCATAATTTTAGGCATCAAAATATTTATTTTGTTAATTTTGTGTAACAAATCATTCATCTGAGAAATATTCTGAATAATCATTTAAACAACTCATAACATTTTTTCAGC

>R=956_68_0_0_0_CG_33_bps_377

CTATGCTCTAATTTCAATTGTGGTATTAATTTCAAATCGATCCAATAAATTTTTGAACTGTAAATGAAGCGATTATGTTACCGCTTGTTAATACTTTAAGTCTACGTCTTAACCGATCAGGCGGGTTGCCATCTGGACGTTTAATATCCTCAGAAATCGTTTCAATGAAATCTTAGGACACATATCCTCTGGAAGCATTTGGTGTTTGGAAAATACCGCAGATCTATTTTATGAAATTCAGAAAAACAACAACTACAAAGGAATTCAAGAAATTAATATTTAAACAAGCTTTTATTATTGAATTCGAGCTCTCACGTTATATGGCATAATAATGATTGAATGGAATGTCATGCAGTAAGTAACGCTGCCATGGCAAG

>R=972_596_0_0_0_CG_35_bps_370

AAAATCAATCAGAATTCAAATCAACACTAAATGAGCATTATGTTTAGTTAATAAGGAAACAGATTATCAGCTAATTAGAAACATACACTATTGAATGCAGTTAGCAAATGATCATTTATCGTTAACTGTCGGACAAATTCCCTGTCAGATTATGGAAATTAAAGCTATGTTCAAACAAAATTTCCACAATATCCAACAAATAGGGTGACGAAATCCTGTAAGTAAAATTAAAGCAATGAAGAGGATATAAGTTAATAATGGACAGCGCAGAACAAAAACGTCTCAGTACCTACACAATGGACAGTGCCAAAGGTTAGCCATACCTCCATGATAGCCCCGATGAAGCTTACAAAGCTCCTCCCTGATCCTT

>R=983_52_0_0_0_CG_26_bps_316

TGTTATGGAAATTTTCTGGTTTTTCGTTTTTGTGGCGTTTCAATTGCTAATGCGAACGAATTGAGATATGTTGGCAAAACTATTTTTGAGTGACAAAAAATAAAAAATGTTGAAAAATGGAATTGCATACAATGGAATAAGTATATGGAAATGGATAAAAGAAAAAGAAAATTGCACATGACATGATTCCGTTATGATAGCCGACATTTCTACAGTAATCTTCTTTTGGTTTTTACTATTAGAAATTTATTTTTCTTCTTGATAAAATATGAGAAAAAAGAAGAAAAAAAGATTTACATAAAACATATGTTATTAA

>R=1001_78_0_0_0_CG_30_bps_661

TGTCTGAAATATCAACGTCATACATTTGTATTATAATTGCATGACAAGAATTTCAATCACCATGGAGAATGTGTTAATTTAATGAAAATATGAATTTAAATGCGAAATAATGTTCTTGTTTTAGTGCACTATCTGTCGGAAAACCCGAGAAAATTTGCAATGCGTTAAAACAATCAAAGTATCACAAATTTGCATCCTAAATATTACCTTATTTAGATCACTTTTGGATAATGGTTTATTATACAAATATTACAATTGAATTTTTAACGTATTAAATGATGGGTCATTTATGCATTTCTCGTAACACTATGGGAAAAGGACACTAGCTTAATAACAAAGTTATTGCAATCTTTGGTCCTTAGTGTACATCTTGGTATGACAATCCAGCTTATGCAAATTGATGGGTATATTGTAAGAGTATCTAATCTGATATTCAGTGGTGCTTATATCCAGTGGCACTCCTTTTGTTCTAGAATTCCTTTGTTTGAGAATTAATTTTTTATGTAATATATGGGTTTTGTAAACCACACCCACAATATACAAAGTTTGAAATCACTTTTTGCTTTACAGGCTCCGACGATCGACCGTATCCTTATTTCCAGCTCCAATTTGTCTTCAATTATCTCTAAACTAAAGCTAATTCAAAGTACTAAAACAAAAC

>R=1009_82_0_0_0_CG_35_bps_483

AGAAAAAAATCTGTTATATGACCCGAAATCCAATGAAGTGAGTTAATTAAAGCTTAATACATCTAATGTCACATGGCTTAATACACAGTATGTGACACCCCGAAGCATTATGTTTATAGCGGAGTGAATTTTGCATAGAATTTCATTACTAGAGCTTATTAGAAGTTCGATCTTCACAACCTAATGAGATAATCGTAAATCCCAGAATCCAGCAAAGTTGATCATTATGATTAGTAACAATCTGAAAAATACTGATGAAGACGATAAAAGTGAAGCACTGGATGCAGCCAAAAAAATTCGAGAAAAAACTGGTAGCGCGCCAATTTGCATTGTTGATCAGCCGGATAATCAAAAGTAAGTCAGACTTGATTTTTTCTTGGCTAACAAATCAATCTAGAATGGAGTAACACCGAGAAGAGCTGATTACGACAATGTTCATCCACAGAATGTGTTTGATAGGATGAACATTAATTTCGGTATCGA

>R=1010_110_0_0_0_CG_26_bps_334

ATATACCACAATGTTGTTTAATGTAATCATCAAGCTTATCCAATATCCGATGTGTAATATTTGATATATTCAATACTTATTCAGCTCAAAACGTGCCATAATATTATTCGATTAATCGCCAGACTTATTCAATATTCGGTATTCGATTTTGATATATTCAATACTTATTCGATGCAAAATATGCCACAATAAATATAATTGTCAAAGTTATTCAGTATCCGATATTCGATATATGATATATTCAATACTTCGACACAAAATATACCACAATATTATTTCAATGTAATCGTCAAACTTATTCAACATTCGATATTCGATACTTGATATATTCAAC

>R=1041_276_0_0_0_CG_32_bps_469

CAACATAATTTTGGTTGCTTTTATTTCTGACACAAATGCAAATTTACTTAATTCAACGAAAGACTTCCAGCGACACGAAAAATTCTGTTAATCCCAATTTCTACAGAAAAACTGTTTGTGACAATTCAGTGAAAGGCTTCCAGTGACACGAAATATTCTGTAAATCCCAATTTCTATAGAAAAACTGTTTGTGACAGTATCATTTGCTTTTTTTCCATAATCAGTATGGAATAATTTTGTTTTACAATGTATTGCTATTCAATACAAATTGGAAAACGGATTAATTTCTATTTCCCGTTGTTTTTCGGAAAAGTTGTATGGAATCTGTTTATCGCAGGTTGCCTACTCCTCAATGTCATATATTTTCTGGAAAAGCATTATATTTGAATTCTTAGCGTTATGCTGATTGTCTTTTTTAATTCCGGACAAAATTCCCGATTCATCGTCCTGGCAGTTCGAAAGACATACA

>R=1043_54_0_0_0_CG_30_bps_496

CAAACTAAACAGCCAATTTTATGTCTTGAAAACAGCCATAATTAGGCATTTGATTAATGAGGCCAGCGATGAAAGTACGTTAGTTGACTGTGTCAATGAAATGACAATAAATATAAAAATTTTCTGGACAATAAACAAGATTTTCGAAATTTTTTCCGTCGAATTAATTGAATTGCTAGAAGATTTGTAAACGGATCAGTTATCACTAGTTGTAACAAAAGATCTGAAACAAAATGGATGACGCGAATTCTATCGTGTTTATTTTACCTTATCACTAATGGTAGTTCCCTTATTGTTTAAATTTCCAGAAACCTAAAATGTAAATATAACAACATACGCAGTAAGCGGATTTATTCATCCGAAAATTACCAACTTTATAAATAGCATGAATATAATGAATTATGCGTGTTACAAAATATGCAAAAATCGATATATCTAAGGAAATGAATGGAATCCCTTGGAGTAATGTCATCGTAACAAACTATACATTACATGA

>R=1052_94_0_0_0_CG_24_bps_598

GCTTAAGAAACTGGTTCGATTATTATAAAATATACACAAAAAAGCAAGTAAAAGGCATTTAAAAATACAGCTAAAAATAAAATGCTTTAAAAAATAGAATTGGTGATAACTTTTTCATAAATAGAGTAAGTGAAAAGCATGCAAAGTAAACTTGGCAATCAAATATGCAAAAATGAAATAAGTAAAAGGCATGGAAAAATAGAGTTGGCACCAAAATATGAACAAATAAAGTAAATGAAAGGCATGCAAAAATAAAGCTAGTAACAAAGTGCGTAAAACAAAGTAATGAAATATGTACAAATAAAGTTAGTAAAAACATGTGAAAATAAAATAAGCAATAATGTGTACAAATAAAGTAAGTAAAAGGCATGCAAAAAATACAAGGAATGAAAGTAAAGGACATGCAAAATAAAGTAGGTAATAAAATATGTACAAAAAAAAGTAAATAAACGATATAGAAAAATAAGTAAGTAATGACTCGTATAAATAAGGTAAATAAAATAATATGCGAAAATAGAGTTGTTAATAACCCATGCACAAGGAAAGTAAATAAAATATGCAAAAATAAAATAAGCAATGATATGTACAAACAAAGCAA

>R=1328_164_0_0_0_CG_30_bps_314

TTCAAGGAAATATTATTATCCTTCCCAGCGCTCAAATCAAAGTACGTTTTTTCAAAACCTCTTTCATGTTTTCAGACCACGTTCCTTTATGCTCAGTCGAGTCTTTTTGTTGAATCCCGAAATTTCATCGAAATTTGCATCTTAAATTGTTTCTTCAGTTTCTAGGAAATTGACATTTCAATTCTCTCCTGTCTCCATTTTTCAGCGATATAGATTTATTTGGAATGTAATATTGTTTTTGCTTAATATTACTATTTAATAACAAAAAAGATGTATACATGTATATATTACGCGGTGAATTGATGAGCAATGAT

>R=1338_52_0_0_0_CG_26_bps_409

TGGCAAACACACCTAAAAATTCTCATCTAAATGTTTTGGATTAATGTAAATTTTCAAAAGATCAGACTGTTTGGGTTAAAGCGTGATAAGGTTTTTGCCATTTTTGATTAGTATATTATCGGTCTGATATATTGATTACTGTCAGATATCACTGTTAAAAATCATTAATCATGTTGTGCACTACCGAACATACATATCAATCATAATTGAATGGGAATTAGCACAAAAAATTAATAATCATTATTTCTAAATTTTAAAAACAGATTAATGCATAAGTGAACTGTAGTAATTACTGCATTTGTTCTAAGTAATATCGTACATTTAAGAAATTTTACATATTTTGGGGGAAATGGAAAAAAAAAGAAATTAATAACATACTGTTGAAATTCTATTGGTCTAAAAAGTCCGT

>R=1444_144_0_0_0_CG_32_bps_438

TTGTTCGTTGTGAAAGCTGTTAAATTTTAAAGAAATGACTTACAATGTATCCTTTATTATCGTCTGGATACCAAATAGAGTGAAGATAGGTTCAAATAATCCCAATTACAATAGAATGACTTCCAGGGTAAAACCAGTAGAATAATGAGAGATTTTGTGAGGATTAGTAGATTGGGAGAGATAGAGGAGAGCATTTGTGTGAACCTGAGTGTCGACAGATGGGTTTTTGGCAACAATGAATGAATCTTAGGATTCCTTATGTTTATATATGGAGTGTGTAAGGAGAAACAATCATAACAAGTTAACGTATACTCACAGCTACCAGAGAAATTACCATATATATATTAACTTATATTAAGTGGTTTCAATCACAATATACTTTGTTTGGAGAAAGGAAAGAAACACAATAATTAACAGAAATCTAGAGATAAACATGTT

>R=1456_242_0_0_0_CG_38_bps_353

CATTATTGTAAACTTTTTCCAATCTGTTAATCAATTTTATATGCAATTGTCGGAATGTTGTAAAGAACTTATATGCAAATGTTGAAATTTTATAAAGAACCTCTAGTCTGGTAGTTAAACCAATTTGTCTGCGATGTTAAACCATTCCCATTATAAAAGGAGAGCTAGGATCAATCATCCGGACGAGCAGTTACAAAAAAGCTTGAGTAGTCACTCAATCCGACAACTCTCACAGTTTCGCAATCCCACAGATCGCTAAGTTACTCAATCGGAGAGCTTGCACAAGTACTCAAGGAGACAATACAGTACAGTTAGGCAATCAACCATGACAACGGTTTAACGGCCGAGAAGCG

>R=1491_286_0_0_0_CG_27_bps_307

AATATTGTACAAGAACAATATTACCTCCCGAATAAATCTGTATCACTGAGAAACGAGTTCAACAGAAAAACAATTTCCTAACATCATTTGCATTAATATCACCAAATCATTCAAAAATGAATTTCTGCCAAATGTTTAACAAACATCAAATCAAACTTTTTAATGTTCAAAAACAGACGTTTTATTAGCAGATTTTCAAAATTTATCAAATCAATTTATAATTCCACTTCTATAAACTTCCACAGAAATCCTTTGTTGGAAATAATGTAATTCATCGAAGTATGCACAGATAAAAATTCGTTGGAAA

>R=1546_132_0_0_0_CG_26_bps_369

GTTGAATATATCAAGTATCGAATATCGAATATTGAATAAGTTTGACGATTACATTGAAATAATATTGTGGTATATTTTGTGTCGAAGTATTGAATATATCATATATCGAATATCGGATACTGAATAACTTTGACAATTATATTTATTGTGGCATATTTTGCACCGAATAAGTATTGAATATATCAAAATCGAATACCGAATATTGAATAAGTCTGGCGATTAATCGAATAATATTATGGCACGTTTTGAGCTGAATAAGTATTGAATATATCAAATATTACACATCGGATATTGGATAAGCTTGATGATTACATTAAACAACATTGTGGTATATTGTGTATAGATAAGTATTGAATATATCAAATATCG

>R=1610_464_0_0_0_CG_32_bps_356

ATATTACTAGTTAACAACAAGAAAGGTGTAAAATATATATGTATATATTACGCGGTGAATTGGTAAGCAGTGATAAATGAGTTATATAATAAGCAAGCGTTGTGATTAACTAACAAATTCCTTGTCGTTGCTTCGATGATAGTTTGATTATCGTTTTATTGATAGGTTACGTAATGGTTGAGATATGAGAATTCGTCCTTCGTTGAACTGGGGATATGTTGCAGGAAATAATTTTAGCGAAAAGGAGAGATACTGTAGTCTGTTGTTCAATGATGGTGGTGGATATGCGAAGAGGATGTTGCAGTTTGCTTTAATAGTAAAAAAAAAAAGAAAAACTGAAGTTTCGAATTTTTCCT

>R=1628_698_0_0_0_GC_33_bps_501

TACACAATGAACAATGTCAAAGGCTAGTCATACCTCCATGATAGCCCCGATGGAACTTACGAAACTCCTCCCTATTAAGAAGTAATTTCTAATATATATATATATGAAAAAATCCCTAACTGGGCAGTTTTACAATCAACCAGGCAATCCTCTTCGTACATCCTCCACAACTGAACCACAAACAACGACATCTTCACATATGCGCCACCACCGGACAGCAAATTGCGATATTCTTGCTATTCGCTAAATACATTTCCCTTAACATATTCCCAACTCAATGACCTATCAATTAACCAATTGTCAAATTATTATCCAAGGGAATGACAAGGAGAATTTGTTAGTTGATCATATTACTTACTTTTTGTATAATTCATTTATCATTACTTATCAATTCATCGTGTAATATATATTTTACATCCTCTTTTTTGTTGTTAACTAATAATATTATGCAAGAACAATATTACATCTCAAATAAATCTATGTCGCCGAAAAACGAGTTCA

>R=1804_158_0_0_0_CG_34_bps_356

AACAGAATATTTCGTGTCACTGGAAGCCTTCCATTGAATTACATAAATTTGCATTTTTGTCAGAAATAGAAGCATCCAAAAGTATGCTGGACCATCGATTGTCATTATTTTCCATTCAGGGAAAGTTGTAGAATTTACATCATTGTCTATTATTTCTGCCTTTTGTATTTTAATATAGATTTGCAATCCTACAAGCCCTTCGAACTTCCAGAACGATGAATCGGAAATTTTTGTCCTAAACTGAAAAAGACAATCAGTATTCCGCTTCAGAAAATGTATGACATTGAAGAGTAAGCAACCTGCGATAAACAGATTTTATCCAACTTTTCCATCAATGACAGAAAGCTGGTCGAATT

>R=1880_76_0_0_0_CG_31_bps_347

AATGGAAATCACCGCAGTATTTGTACCAAAAGCAATTAAATCTCAATATTGGATGACTTTGGCATTATTTGATAAAAAGATGACTAACTTATAGTAGGAATCCAGGAGAAAATGTTTCATGCACGATGAACTTATACTGTATGGGATAATCACTCCGTTCATTGGACTTCCTTCTATTTCATTAGGTGAGTATGGCCCAGTTGCTGAAATCACATGATTAGAACTATTCGTTTGTTTAATAATGGTTTAATGCCATTCATTGAATAATTACAATAAGATTATAATGAAAAAATCCATTATAAATATCTAACAAGATGCGGATGTTTCGTCGATAATTTCTAGGTGAT

>R=1895_304_0_0_0_CG_35_bps_568

AGCCGAGAAAATGCTATGAGTTGTTTTAATTAGAATTCAGAATATTTCTCATACAAATGGTTTGTTACAGAAAATCAATAAAATAAATATTTCGATACCTAAAGTGATGAATCATTCATCCAAATCCAACTCTTTCAATTTTTTAACCAATCTTAACTGCAATGTTTAAATGTTGTGGACAAAACCCTTTGTTGTGAATCTTTTTTTTTTTCAATCCATTAATCAAGCTTACATGCAATTGTCGGAATGTTGCAAAGAACTTATGTACAAATGTCAAAAACCTCTAGTCTGCCAGTTATCTGGAATGTTAAACCATTTCCACTATAAAAGAAGTGAGGATCAGTCATCCTGCCGGTCAATTACCAGATCAAAAAGTTCCACCAGTCATTCAATCCTACAGCTTCCCACATTTACTCAATCTGACAAATCCCGCAGTTACGTAATTTGATAGTTCGTTGAACGGACAATCAGACAGCTCGTGTAAGCACTCAAGGAGACAACAAAGTTAGGAATTCAACCAGGACAACGATTTAACGACAGAGAAATGGCAATACGACAATTCAGCAGC

>R=1948_90_0_0_0_CG_30_bps_595

CTTCACAATTATCCTTATTAAATTTTCTCTTTCCCTTTTTCTTCCTCTCGAGAATGTTGTGAAAACGAAATTGCTAATGTTGAAAAACAGAATAAAATTATATGTTAAACATATGGTATTTGTATTTATTAATCAAAGTGGACATGTAACAATAGCATATGAATTGAAAATCAAATACTATCCAAATTAGTGTTATCAAACATCAATCGCAGAGTAATCTTTGATACCTGTCACATTAGTCAAAGATTAATTTCCCTCGTCAATCACAGTGACTCCCTCAGGATATTATAAATGATCAAAAGAGCCTACAATGCATCATCCAGCATAAATTTCTGATGAAATCGGAAATATACATAAATTTGACTTCTTCAATAATTTATTATGTTATTTTTAAAATGTCATTAAGCATTTATCTCAGATTATTCTTAATATGGCTCCCATATTCGGCATATTCCCTTCCCTATGTTAAAAGATCATCGATTTAGCAGCAGCAGATCGAAGAATGAAAGTGAGAATTTGTAAGAACAATTGCATCTCGAGATGGAAGTGATTTCTTTTCAATCGGATCCGAATTTTATTATCGTGAAAGATTTTT

>R=2050_98_0_0_0_CG_32_bps_416

CAAGTATTATGACCATTCGACTGTCACTTTTAATTGATGCAAAGTTGGAATGGAAATCACCGCAGTATTTGTACCAAAAACAATTAAATCTCAATATTGGATGACTTTGGCATAATTTGATAAAAAGATGACCAACATATAGTAGGCATCCAAAAGAAAATGTTTCATGCACGATGAACTTATACCGTATGGGATAATCACTCCGTTCATTGGACTTCCTTCTATTTCATTTGGTGAGTATGGCCCAGTTGCTGAAATCACATGATTAGAACTATTCGTTTGTTTAATAGTGGTTTAATGCCATTCATTGAATAATTACAATAAGATTATGATGAAAAAATCCATTATAAATTTCTAACAAGATGCGGGTATTTCGTCGATAATTTCTAGGTGATCTTTGTGAGATGTGAATATCG

>R=2194_104_0_0_0_CG_44_bps_326

GTCGGAATGTTGTAAAGAAATTATGCGCAAATGTCGCAACATCGTTGAGAACTTCTAGTTTGGTAGTTGAATCAATCTGTCTGGAATGTCAAATCATTCCAACAAAAAAGGAGCTAGGATCAGTCATCCCAGCTCCTTCAGTTATCCTAGCTCGTTTACCAGATCAAAAAGCTCGAGCAATCCGACAGATCGCTCAGTCACTCAATCGGGCAGCTCGGGCAAGCATTCAAAACGACATATTGTCAGACACTCAATTTGACAACGGTTTAACGGCCGAGAAGCGGCAATACGACAGTTCAATAGTACGAGCGACAAGACCGTTTGGT

>R=2335_184_0_0_0_CG_29_bps_429

AAGTTATTGTAAGTTTGTTGCTAATTATTAATTAACTTAAAATGAGTTAGTAAAAGGGTTTTTAATATATTTATTAGAAGTATGAAACGATTAAATGGTTTATCGATGGTGCTAATGCTAATGGTCTTGAAAAGCTGCACCCTTTTCATCGGCTAACCAACAATGTTTCATTGCTAGTACTACATTTATTACTACTATTATTTACTAATTTACTCTTCGATGCTTGATGTTCCAAATAGAGCCTTTTGTTGTAATCGGATTTCGACAATTTTTCATTATCCAAAGGAAAATCTAATGTAATATTTGTTATAAATCACACCCGCAAATATGTTCATTGTGTTAATTAAAATTGTATATATAATCAAGGCTATAACTGAAAGATCATTTTTCCTTTTCAAACACTCAACCAAGGAGTGCACCGTACATTGT

>R=2352_274_0_0_0_CG_32_bps_316

TTGTTAACTAGTAATATTGTGCAGGAACAATATTGCATCCCGAATCTATATCATTGAATAACGAATTCAACAGATTAGCTTTCCCGATACTATAATAATTAGTTTCAATTAAAAGTTATGAAGGAGTGGTAAATGATATGACAACACTAATGTTAATGATGATATCATTAGTGATGTCTGTAGTACTTTATGATATTTGTAGATATGGCAGTGATAACTTTTCATGTTCTGCTCAATTGCTAGAGGAAAGAGGACTCTTTTTTTTCTTTTCGCCGAATTTTCTCCTTCAATGCAATTATCCACATCACTGCCATCG

>R=2372_170_0_0_0_CG_31_bps_357

ATCATTGCTCACCAATTCACCGCGTAATATATACATGAATACATCTTTTTTGTTATTAAATAGTAATATTAAGCAAAAACAATATTACATTCCAAATAAATCTATATCGCTGAAAAATGGAGACAGGAGAGAATTGAAATGTCAATTTCCTAGAAACTGAAGAAACAATTTAAGATGCAAATTTCGATGAAATTTCGGGACTCAACAAAAAGACTCGACTGAGCATAAAGGAACGTGGTCTGAAAACATGAAAGAGGTTTTGAAAAAACGTACTTTGATTTGAGCGCTGGGAAGGATAATAATATTTCCTTGAATTGTTCTGGATGAGAAGGACCTACTATATTTAACCTATTAAGT

>R=2639_54_0_0_0_CG_35_bps_420

AAAATTTTATTACCTTAGTATTTGATAATGAAATTAGAAGATGGCATACGGAGTACAGTCAATTTAAACGAACAGTCAATAGTCGAGTTGAGCAAGACGAAGATGGCCCTGATTTGATCTTCAAACAAGATTTTCTTTAAGAATGCATTGAATCTCCTTCTTGGCGCCTTTATATTGCCCATAAGTGGGTTGGGCTTACGACAAGGGAATAACTTCAAATAAGGTCTTCACAAAGCTGATAATAATTTCCTGGTGCAAGAGAAAATTGTATAAAAGACTAGATTTCTTCTTTATATGAAGAGGAAATTGCTGGCAATCATCTTACTAGACGTACAGCTTTCTCATAGTTTGTATGGAAATTAGCTAGCGGATGTGGGTAATTTCAATATCAAAGGAAAGGACATTTTTACACGATTGAGT

>R=2785_112_0_0_0_CG_31_bps_332

TTTGATGTAACAAAATACCAGAAATACAGATAGTATCTATTAACTAAGTAGAAATTAACGCAAATGAGATGCAATCGTTACTTTAATTCCGAGGAATAGATTGAAATGAATTATTGGGAGGATAAAGTAGTAAACGGAACAAGATAACAAGCTGGATCGGTGATTTAAATGAAGTAGTAGAAATTCATTTGCAGGGATGAATGCGTATATAGTGTTTATTGAGTGAACAATGAGTACCAATATCAAATCCTAGTTAATTTATATTGAGAACATGTGAGTGTGGATTACAATATGAGAATAGCTCCACATAAGTCTTTACAAATCTGACAGTA

>R=2844_92_0_0_0_CG_32_bps_388

ATAATACCAGTATAACAATGGGCGAAATTTGTAAGTAGTAACGTAATGAACTGGGAGAAATATGAAGAGTGTGTTGTGGGAGGCTGAGTGCCGACCGATAGAATTGAATGAATTGATGTTAGAATTTCTTATGGTTTATATAAGGAAATGTGTGAAGAGAAATTTTGTAACAATCATTAGAAATTGACAATAGTCTCACAGCTACAAAAAATTTACATACCAGGCATAAGATTGGTTTTATAGATAAATAGGAAATTACAGGAAATTATTTTGTGAGATGTACAGCTTCCTGAAGCAGTATGCAAGATTTGTGCATAAGATAAGGCTTTTACAAATTGTAGAACGAGACAGGTAACATTTTTAGACGATTTGAAGATTAGATAGTTAA

>R=2980_208_0_0_0_CG_33_bps_381

CAAGGAAACATAAAGCGTTTCCCATAACCAGAGTTAAGAGTCATCTAACCTTCAAGTCGTTTAAAAATGCTTTGATGTTGAGAGATGCGTACATCCGGTAACAAATTTGTAAAGGCTTTATCCTGTGTACAAATCATAGATATAGCTGTGAGAAAGTTGCACATCCAGTCAATTTCTATGCAATTTCCACTTTATATGAAGAAAACTGATATGTAAATTTCTCTTGCAGCTGTGAAAATACTGTCAGTTTTATAAGGACCTTATAAGCAGCTATTCTCATATTGTAATCACACCCACTTATTCTCAATATGAATTAATAAGGATTTGAAATAAAGACTCGTTGTTGATTCAAGAGAAATACGCAGTCATATCTGCAAAGGA

>R=3065_52_0_0_0_CG_33_bps_664

AAAATTGAGATAGATGTCCTGGTAGTAAGAAGCGACTAAGTTGGTGACAAATGATTAAGCATAATTATGGGAGGGAGAACGATGCTGAGACAAAATGACGTGGTGGACATAGTTTGGGAACAAAATACCATAAATCGGAAGACTTCATAGAGGAAATGAGTTAATAAGTTATGCAAGGGCATCCATTGTAGACAGGCGATTCATTCATGACCCTGGATGACCTCATCAAATCGTTTTTTTCCTATTCCCTTGCTTCGATGGCGATCATTTCTATTCTTTCTACACAGTTCTAGAGAATTGCTTTTTCTCAAAGTGATCTTCAGAGTTATTTCAATTTTTATTAAGCAGTGTTTCACTTTAAATTTTCCAAAATTAAATATATCAACAAGGCCAAAAAATCAATTATTAACTTCCAAACTCTGACTTACAACGATTTAATTTCATTTATTGCTATTTTAAGCTGTGGTCGCAAAGTTTCAATACGAGGGAATTACTAGCACAAATTTCTACTTCATTTCGACTTTTCAGCTCATTTTTCAAACCATTTCCACGAGATTTAATTTGGTTTAACTCCGATACAAATATGCAAACACATCTTTAACTATCAAATCGAGATGAGTCATATTCATGAAATACCATGATTTGACATTATATTGCAAATTAG

>R=3469_82_0_0_0_CG_32_bps_359

CCATATCATTGCCGATCAATATTCAGTAGATCAACAAGATCATTCAAGTGATGAGCATCCTTCAGAAAACTTTATCAACCTCGACAAGCCTGATGATAGCATCTTCGATGATCTTAACATCCCCGATAATGTTGTTGTTAACATCGAAGATATTAATCTTGAAAACTTTGACAACGACAACCTTAATGGTAACATCGTCGACGATATCTCTCGTTTCTAAATTTTATAGCAAAAAAAAGAAAACTTGGTCATGAAAATCAACTTTCTTTTTTGTCTTGTAAGAATTATCTAAAGATCTCATATAAAAAAATTTTCCATTGGAACTTTGGGAACATTTAACGAACACTTACATTTCTTGA

>R=3695_148_0_0_0_CG_35_bps_342

CTTTATCGTGATAAGAGATTGATAAAAATCCGCCAATGTCCGTAGAAGCAAAGAATAAACAGAAGAAGAAGCCGATAACCTTCGAAGAGGAGAGCAACCAAAAAACCCCCCCGGATTTGGGACCGAGATTACATATGTTAAGAAATGGGAGTGGCTTACATGAAGAATTCAAGTATCGGAAACAAACCGGATATGGTACAAACATTATAACAAGATTAAACAGAATGATTGACATGGGAAATTAAGGGTTGAAACATAAAAATGCAAATTGGTTAGATAAGATAACAACAAAAGAGTGTAGGAAACAATTTTAAGGAATGGGAAAAAATTTTAAGGAATAAG

>R=3790_62_0_0_0_CG_35_bps_363

AATGCTTCGTTTTGTGTTCTCCTCCTCGTTCTACGGTCTAAACGTCGCCTTAATTATGTTTTTGTCCAAAGTGATCCATTTGAAAAGAAATCACTTCCTATCTTTAGATACAATTGCTCTTCCAAATTTCCGCTTTCATTCTTCGATCTGTTGCATATAGATCGAAGATCTTTTAACCGATGGATTCAGAATTTGAATGACACATAGGCAATATAATGCATATTTGAGAGCTCAAATGTAATATATTTGCGATTTGAACGACACTCAAAGGAACATGTACGTGTGCTTTCAAAGGAACGTGTGACAAAACGACAATATCTAATGCATATTTGAGAAGAGCCAAAAGTATAATATACTCACACG

>R=3984_60_0_0_0_CG_30_bps_515

CAAACTAAACAGCCAATTTTATGTCTTGAAAACAGCCATAATTAGGCATTTGATTAATGAGGCCAGCGATGAAAGTACGTTAGTTGACTGTGTCAATGAAATGACAATAAATATAAAAATTTTCTGGACAATAAACAAGATTTTCGAAATTTTTTCCGTCGAATTAATTGAATTGCTAGAAGATTTGTAAACGGATCAGTTATCACTAGTTGTAACAAAAGATCTGAAACAAAATGGATGACGCGAATTCTATCGTGTTTATTTTACCTTATCACTAATGGTAGTTCCCTTATTGTTTAAATTTCCAGAAACCTAAAATGTAAATATAACAACATACGCAGTAAGCGGATTTATTCATCCGAAAATTACCAACTTTATAAATAGCATGAATATAATGAATTATGCGTGTTACAAAATATGCAAAAATCGATATATCTAAGGAAATGAATGGAATCCCTTGGAGTAATGTCATCGTGACAAACTATACATTACATGAGAAATGAGAATATTAAATG

>R=4047_118_0_0_0_CG_29_bps_397

TCTGATATTTTTATTTGTAGCAAAGGCAACATAAGGTTCAAAAATAACAATTTCTGTTCATTTGGAAATAAACTTTAATTCACACTTTTAACATATTAGTTTACCAAACATTGATAAGAGCAATTCCCGGTTGTTTTCTACTCACCGTGCTGCGCCTTCGCGAATCATCTATGATCCGTGAATAACCGGTGGTGAACCGCAGACTAAAAAAAAATTTTTTTTCTCTTCTATTTACCTAAAGATTTTTATAAATTTGAGAATCTTATATGTTGTATTCATTCCCAAAAATTTTCGAATGTTTTATATTAAATGACAAAAGAATGCATTCACGGGGAAAAAAAAGAGAAGCACGAAGAAGTAATAAAAATTAAAAAATCAAAGTATGCGAAATAATATC

>R=4062_286_0_0_0_CG_28_bps_492

AATATTGTACAAGAACAATATTACCTCCCGAATAAATCTGTATCACTGAGAAACGAGTTCAACAGAAAAACAATTTCCTAACATCATTTGCATTAATATCACCAAATCATTCAAAAATGAATTTCTGCCAAATGTTTAACAAACATCAAATCAAACTTTTTAATGTTCAAAAACAGACGTTTTATTAGCAGATTTTCAAAATTTATCAAATCAATTTATAATTCCACTTCTATAAACTTCCACAGAAATCCTTTGTTGGAAATAATGTAATTCATCGAAGTATGCACAGATAAAAATTCGTTGGAAATTAATTCGTCTATTTGCAATAAAAAATGGAGAAAAGGAAGTAGTCATAGAAAATACTTCAAACGATTGTAGAATTGTTTTTACAAAAAATAAAACAGTTTTAGCATTTTGATGGAGACCGATGAAAAACAGTTTTCCGACAATTATCGAATGATTGCTGTTGATCTTTAATATCTCGACCACAAT

>R=4260_154_0_0_0_CG_33_bps_308

CCCAGGATTTGGGACCGAGATATACATGTTAAGAAATGGGAGTAGTTTACATGAAGAATTCAAGAACGGAAAAACACCGGAGATGGTACAAGCATTATAACAAGATTAAACAGAAAGATTGACATGGGAAATTAAGGGTTCAAACATAAGAATGCAAATTGGTTGGATAAGGTAACAACAAAAGAATGTGGGAAACAATTTTAAGGAATGTAGATATGTTGAGAGTGATTCATAGTATCATTAGCATTAATTTACAATATGAATGAATTTATTTGAGGTTTCGTTTTCACGACAGGGAGTACTTCAAG

>R=4451_154_0_0_0_CG_34_bps_329

CCCAGGATTTGGGACCGAGATATACATGTTAAGAAATGGGAGTAGTTTACATGAAGAATTCAAGAACGGAAAAACACCGGAGATGGTACAAGCATTATAACAAGATTAAACAGAAAGATTGACATGGGAAATTAAGGGTTCAAACATAAGAATGCAAATTGGTTGGATAAGGTAACAACAAAAGAATGTGGGAAACAATTTTAAGGAATGTAGATATGTTGAGAGTGATTCATAGTATCATTAGCATTAATTTACAATATGAATGAATTTATTTGAGGTTTCGTTTTCACGACAGGGAGTACTTCAAGAGCGGTACCTCACTCAATTAA

>R=4473_62_0_0_0_CG_43_bps_311

AATGTTGCAAAGAACTTATGTACAAATGTCAAAAACCTCTAGTCTGCCAGTTATCTGGAATGTTAAACCATTTCCACTATAAAAGAAGTGAGGATCAGTCATCCTGCCGGTCAATTACCAGATCAAAAAGTTCCACCAGTCATTCAATCCTGATAGCTTCCGCAATCACTCAATCCCTTAATTCCCACAGTTACATAATCCGGCAACTCGTTCAATCACTCAGTCGGGCGGTTCGTGTGAGTACTCAAGACGAAATACAGTCAGGCATCCAATTTCGACTACAATTCAACGGCACGAGCGCGACAAGACCG

>R=4529_78_0_0_0_CG_25_bps_545

ACATTCCATTACGTACTTTATTTTCACATACCTTTTACTTACTGTGTTTATACATGTTTTATTACTTTATTTTTCCATGCCTTTTATTCACTTTAACTGTGCATATATTACTCATTACTTTATTTTCGCATGTCATTTATTCATTTATTTGCGTTTTTTTTTTTTGCATGCCATTTATTTTTACTTTATTTTTGCATGCTGATTTATTTGTCCATATTTTTTTTGTGCATATTTTACTACTGACTTTATTTTTTCATGCGTTTTACTCACTCTATTTCTGCATATTTTATTATAAAATTCTTTTCTTTATGCTTTTACTCACTTTTCTTCCGCATGCATTATTAGTACAGCTTTATTTTTGCATGCCTTTTATTTATTTTATTTGTACATATCATTGCTTACTTTATTTTTGCATGTCGTTCAACTAACTTTATTCTTACATATTTCATTATGAACTTCATTTTTGAATGCTTTTTACTCAACTTTTCTCTGCATGTATTATTACCAACATTATTTTTGCATGCCTCTTACTCACCTTTTTCTTT

>R=4564_68_0_0_0_CG_30_bps_518

TTTACAACCTATTGTCAAAGTCATTATCTAATTGATCGGGCAGAATGCTGATGCAGCTCTAACCGACAACAATCCAGAATGACAAATTAATCCTCTCATGGAAAATTCACTAACGACATCATATTTGTATAAGCAAGATTTAAAGGCAATTGTTTATGATTAGAGTAAGCAAATTACCTCATCAAAGCGTTTATCAAAACTAGCTAAAGATATTTTTGATATTTTCGAAATTTTGTCAAGAAATGCAAGTATCCTTCAAATGTGCCAAAACTTCAATGGAAAATCTTTTTATATAAACACTGAAACAATTCATACAAGACCAACCAAAAAAAACAAACTTTTTTTTCTCGAAAACATATTTTGTCGTATATTCTCCTCAATTTCAAGTAAAAGCTATATTTTTAATACACAAATTAGCTGTACCGTCAATCTTATTCAACAAGTTGATAACAAGCAGGTATAAAAAGAGACTCATTATCCGAATGATTTTGTTGACCCAATGAACATTGACCGTGATG

>R=4648_100_0_0_0_CG_31_bps_315

ATTCGTTCTTACTGACATATTGTTCAAGAATAAATTTACTGTATTATAATGAATTATAAGATTAACATGCAGAGTACTTGCCATGGCAGCGTGACTTACCGCATGACATTCCATTCAATCATTATTATGCCATATAACGTGAGAGCTCGAATTCAATAATAAAAGCTGATTTAAATATTAATTTCTTGAATTCCTTTGTAGCTGTTGTTTTTCTGAATGTCATAAAATAGATCTGCGGTATTTTCCAAACACCAAATGCTTCCAGAAAATATGTGTCCTAAGATTTCATTGAAACGATTTCTGAGGATATTAAAC

>R=4702_52_0_0_0_CG_25_bps_432

TGTTATGGAAATTTTCTGGTTTTTCGTTTTTGTGGCGTTTCAATTGCTAATGCGAACGAATTGAGATATGTTGGCAAAACTATTTTTGAGTGACAAAAAATAAAAAATGTTGAAAAATGGAATTGCATACAATGGAATAAGTATATGGAAATGGATAAAAGAAAAAGAAAATTGCACATGACATGATTCCGTTATGATAGCCGACATTTCTACAGTAATCTTCTTTTGGTTTTTACTATTAGAAATTTATTTTTCTTCTTGATAAAATATGAGAAAAAAGAAGAAAAAAAGATTTACATAAAACATATGTTATTAAAATTAATTTTGAAAAGAAAATTTCGACAATTTTTATTGAGTAAATTAATTGAATCATGCAGTATTATTCGAATTTTTTATAAGTTTTCAAGCAAATTTCTTCGGAAAAGTCAATAC

>R=5349_66_0_0_0_CG_29_bps_323

AAAAAGTGTTATGAGTGGTTTTAATCAGAATTCGTTAATTCCTCAAGTAAATGATTTGTTACATAAAATTTAAAAATAAATATGTTAATACCTAAATTGATACACTCTTTTTCGGCAAATGGGTTGGAGAAACCATCAGTCCTATATTCCGGGATGCAATCGTGCATTCCGGATCAAGATTTACTAACATTATTATTCATTTAGCAACATTTCGCATAAGTGTTTTCGCATAAGCAGTTAGTGATATCTAATATGTGTAATATATTGATATGGAGAAAGTTAGTGATATTTTTAGTTAGTGATATCTAATATCTGTGATAACG

> R=5502_140_0_0_0_CG_28_bps_652

CAAGATTGTCTATACAACTGCAGACAAAACTGCTTCAAACCATTATATTGTTTGACCGGCTAGACAGTTCAAAATGATTTTGCAGAAACCCGATGCATTTCAGTTTATTTTTGACCAAAATTATATATAAATAAACAAACGTAAAATGTATGAGACTATTCACCTTTCGAATATTTTTCAAAATATGCAAGCAAATTTAAGAAGCAAACATTACTTTCAGTGGAATTACTCTGATTAATCTCAAATCAACTATTACTGTAGGAAAATCAATATTAAAATTATATAATTGCCCACCTTTGCTCTTTAATCAAATTTTTTTCCTGAAATTTAAATCGTATTGGACAATGGTACAACTTCCATCGGTTTTGGAAGCAATAAAATTACCAGAAAGGAAAATATTGTTATCAAGCATTGAACTTAGTGTCAAGTGTTCAACTTCTTGTGCTTAAAACGATCTAGTCATTCTTTCCCTCGGAAATATATTCATTGCATTTTTTGGAAAAGGGAAAATGAAAAAGAAAAATTGAAATTCTTAATTTTGATCAAAACTTATCTCAACATTGAATCTATTTCAATATTATGACGAACTAATTTGTATAACACATTTAACAAAAAAATAAAAATAAGAAAATGCCTAATTTAGCCACATACA

>R=5587_144_0_0_0_CG_30_bps_622

TTTTTCCATTTCCCCAAAAATATGTAAAATTTCTTAAATCTACGACATTACTTAGAACAAATGCAGTAATTACTACAGTTCACTTATGCATGAATCTGTTTTTAAAATTCAGAAATAATGATTATTAATTTTTTGTGCTAATTCCCATTCAATTATAATTGATATGTATGTTCAGTAGTGCACAACATGATTAATGATTTTTAACAGTGATATCTGACAGTAATCAATATATCACACCGATAACATACTAATCAAAAATGGCAAAAACCTTATCACGCTTTAACCCAAACAGTCTGATCTTTTGAAAATTTACATTAATCCAAAACATTTAGATGAGAATTTTTAGGTGTGTTTGCCAGTACCCCACATGTTCAGTAATTTTTGACAGTAATCAATATCTCACATTGATAACACACTGAACAAAAATGGCAAAAACTTAAAAATTGTCATCCAAGTGTTCGGAGTGGTGTAAATTTTCGAAAAATCTTTGACTATCAGGCTCAGGCTTAGATTTAGGCTTGCTTAATATTTGGTAAGGTTTTGGCCAATTCTAATCAATGTGTTATCGATCTGATGTATTGATTACTGTCAAAAATCACTGATTATCTTGCACACTCGCGAA

>R=5666_54_0_0_0_CG_31_bps_524

TTGATAGAGAAATATTTTCATTTTTTTTTCAATTACTATTTGGTTAAAAAAATTTTTATTTAAATTTTCCTGAATCGAAATTTCAAAAAATTTTATTACTTTAGAAAATTTTATTACCTTAGTATTTGATAATGAAATTAGAAGATGGCATACGGAGTACAGTCAATTTAAACGAACAGTCAATAGTCGAGTTGAGCAAGACGAAGATGGCCCTGATTTGATCTTCAAACAAGATTTTCTTTAAGAATGCATTGAATCTCCTTCTTGGCGCCTTTATATTGCCCATAAGTGGGTTGGGCTTACGACAAGGGAATAACTTCAAATAAGGTCTTCACAAAGCTGATAATAATTTCCTGGTGCAAGAGAAAATTGTATAAAAGACTAGATTTCTTCTTTATATGAAGAGGAAATTGCTGGCAATCATCTTACTAGACGTACAGCTTTCTCATAGTTTGTATGGAAATTAGCTAGCGGATGTGGGTAATTTCAATATCAAAGGAAAGGACATTTTTACACGATTGAGT

>R=5970_70_0_0_0_CG_35_bps_313

CAAGCAATATGATCAATTAAATTCCTTGTGTTGCCATCAGTAATAGTTGATTTTCAGTTTGTTGATAGGTTGCATAATGATTGCGATATAGGAATTCGTCCTTCGTTAAGCTGGAGATGTACTGTCGGATAGGTATTTATTTAGTGGAAGGAAGGAATATTGTAGCTTGTTTCCAATGGTGACGGATGGACGAAGAGAACGCTATGGTCTGCTTGAATTGTAAAAACTGATTTTGTAGGGTGTTTTCCATATATATACACCTAAAATTAGAAGAGAAACTTTAGTAAGCTGGACTGGGTTATCATATAGATAT

>R=6310_578_0_0_0_CG_32_bps_579

TAATATGATCAACTAACAAACTCCTTGTCGTTACCTTGGACAACAATTTGATTGTCGGTTAGTTAATAGGCCGTTGAGTTGGGAACACGTTACGGGAAATGTATTTGGTGAATAGAAGAATATCGTAGTTTGTTGTCCAATGGCGAAAATGTCGTCGCTTGTAGTTTATACGTGGAGGATAAGCGAAGTAAACTCCATCTAAGGTACTTACAATGCTGGCAATAATTTTACTCATACAAGAGTTGCTTACATAGAAGATTAGACTTCTTTGTAAGAAGGAAAATCGCCATAAATCATCTGAACATCAATGAAAAAGATATTTTTACCGATTGACCTTAGACAACAAATTGACCTCTAACAAAGAAGAAATTATTTACCAATTTATACAACAAATTATTTACAACAAATTCTTTAGCTATTTGGACGTATTACAATTTCCTTCAGTTTTTCCCGATACTAGCTTTAAATTTCGGTAATCCGACAGAAAATTTGCATTAGGATTTTCGGGGTTTTTCCGACATTAACAATGATGATAATAACGTCATCATAATAATGGAACGAATTTTCTATTGTATTCTT

>R=6425_146_0_0_0_CG_30_bps_635

TTTTTCCATTTCCCCAAAAATATGTAAAATTTCTTAAATCTACGACATTACTTAGAACAAATGCAGTAATTACTACAGTTCACTTATGCATGAATCTGTTTTTAAAATTCAGAAATAATGATTATTAATTTTTTGTGCTAATTCCCATTCAATTATAATTGATATGTATGTTCAGTAGTGCACAACATGATTAATGATTTTTAACAGTGATATCTGACAGTAATCAATATATCACACCGATAACATACTAATCAAAAATGGCAAAAACCTTATCACGCTTTAACCCAAACAGTCTGATCTTTTGAAAATTTACATTAATCCAAAACATTTAGATGAGAATTTTTAGGTGTGTTTGCCAGTACCCCACATGTTCAGTAATTTTTGACAGTAATCAATATCTCACATTGATAACACACTGAACAAAAATGGCAAAAACTTAAAAATTGTCATCCAAGTGTTCGGAGTGGTGTAAATTTTCGAAAAATCTTTGACTATCAGGCTCAGGCTTAGATTTAGGCTTGCTTAATATTTGGTAAGGTTTTGGCCAATTCTAATCAATGTGTTATCGATCTGATGTATTGATTACTGTCAAAAATCACTGATTATCTTGCACACTCGCGAACACACAAAGAATT

>R=6482_270_0_0_0_CG_29_bps_373

TGTGTCGTAAAATTATTAATTCAAACAATTTGCATTTACATTTTTTTTCTCATGCTTAAAATCATGAGTTAGCTATCTAATCTTCAAATCATCTAAAAGCGCTTTGTCCGATGTTGAGATTTGCGCACATCTAGTAACTAATTTGTAAAAGTTTTATCCCATATGCAAATCTTAGATGTAGCCGTGAGAATGCTGTACATCTGATAAAACAATTTCTATGCAATTTTTTATATAAAGAAAACTCTTCTTATGTGTAAATTTCTCTTACAGCTATGAGAATACTGTCAATTTGTAATGAATGGTCCCTTTCAAAAAATTTTTTATATAAAACCATAAGAAATTCTATCATTCATTTCTGCGCTCAGCTTCACAC

>R=6520_184_0_0_0_CG_32_bps_386

TTAATTGTGCCTTTTGTATTTGAAATAATACTGCAATCCTGTATGTCTTTCGAACTGCCAGGACGAAGAATCGGGAATTTTGTCCGGAATTAAAAAAGACAATCAGCACAACGCTAAGAATTCAAATATAATGCTTTTCCAGAAAATATATGACATTGAGGAGTAGGCAACCTGCGATAAACAGATTCCATACAACTTTTCCGAAAAACAACGGGAAATAGAAATTAATCCGTTTTCCAATTTGTATTGAATAGCAATACATTGTAAAACAAAAATATTCCATACTGATTATGCAAAAAAAGCAAAAGAAAACGGCACAAACAGTTTTTCTATAGAAATTGGGATTAACAGAATATTTCGTGTCACTGAAAGCCTTTCACTGAATT

>R=6840_94_0_0_0_CG_36_bps_319

TTTTTGAGGGCTGGACTAATATACTTAACTCAGTTCTATTCAATATCGTATCCGGTACTCAACTCAGTTTTACCCAATACCGTATCCGTATACGTTTATATGTACAACCCCTCAAAAAAAAACAGTAAATGAGATATTGTAATGGATTCATTGAGGATGTGAAGAGTCTAATTACATCATTATTTAATGTTAACATAATGAACTTATGATGAAAGATCAACGAACTTTAACATCACAGAGTTTTGATTTAAGAACGGTGGAGGGGAAAAGGGACAGGCTTCTCCCGCGGTTCCGCGAGCAAAAGAAACATGCGGAAAGG

>R=6848_70_0_0_0_CG_36_bps_326

CAAGCAATATGATCAATTAAATTCCTTGTGTTGCCATCAGTAATAGTTGATTTTCAGTTTGTTGATAGGTTGCATAATGATTGCGATATAGGAATTCGTCCTTCGTTAAGCTGGAGATGTACTGTCGGATAGGTATTTATTTAGTGGAAGGAAGGAATATTGTAGCTTGTTTCCAATGGTGACGGATGGACGAAGAGAACGCTATGGTCTGCTTGAATTGTAAAAACTGATTTTGTAGGGTGTTTTCCATATATATACACCTAAAATTAGAAGAGAAACTTTAGTAAGCTGGACTGGGTTATCATATAGATATGGCTGCATGATCT

>R=6989_140_0_0_0_CG_28_bps_666

CAAGATTGTCTATACAACTGCAGACAAAACTGCTTCAAACCATTATATTGTTTGACCGGCTAGACAGTTCAAAATGATTTTGCAGAAACCCGATGCATTTCAGTTTATTTTTGACCAAAATTATATATAAATAAACAAACGTAAAATGTATGAGACTATTCACCTTTCGAATATTTTTCAAAATATGCAAGCAAATTTAAGAAGCAAACATTACTTTCAGTGGAATTACTCTGATTAATCTCAAATCAACTATTACTGTAGGAAAATCAATATTAAAATTATATAATTGCCCACCTTTGCTCTTTAATCAAATTTTTTTCCTGAAATTTAAATCGTATTGGACAATGGTACAACTTCCATCGGTTTTGGAAGCAATAAAATTACCAGAAAGGAAAATATTGTTATCAAGCATTGAACTTAGTGTCAAGTGTTCAACTTCTTGTGCTTAAAACGATCTAGTCATTCTTTCCCTCGGAAATATATTCATTGCATTTTTTGGAAAAGGGAAAATGAAAAAGAAAAATTGAAATTCTTAATTTTGATCAAAACTTATCTCAACATTGAATCTATTTCAATATTATGACGAACTAATTTGTATAACACATTTAACAAAAAAATAAAAATAAGAAAATGCCTAATTTAGCCACATACACGAACACTACTCAC

>R=6996_82_0_0_0_CG_35_bps_335

CTTATTTCGATTATCCGATTGAGCAACAATACAAATTGGTACATCTCGTAAAAACTTTCGAATTTCGTCGGCTACAGTCAGTGCTCCACATTTAATGCCTTCATCAGTATATCTGAGATCGTTACTAATCACAATGACCAATACCACACGACCCTGGGATTCATGATTATTTCATTAGGTTCCCAAGATCGTACTTTTAATAAGCTCTAGAAATGAAATCCTATGGAGAATTCAATTCACTATAAACATAATACTTCGGAGTGTTGCATACTGATGTATGCATTAAGCTTTAATTAACTCACTTTATGGATAAGCAATGTGATGATACATGCCCA

>R=7038_108_0_0_0_CG_32_bps_428

CCAAAGGCATAACTGCAGAGAATGCAAAATTGCGATACCAAAATAATATCAAAATAAAAAAGGGAATCATCAATTTGTTAGGTTCTGTAAATATTTTTGCCGATTTTCTACGATAAAATATTACCAAATTTATCCGTAACATTCAAAAAATTCGGATCCATTTGAAAAGAAATCACTTCTTATCTTTAGGTACAATTGCTCTTCCAAATTTCCGCTTTTATTCTTCGATCTGTTGCTTATAGATTAAAGATCTTTTAACCAATGGATTCAGAATTTAAATGACAAGAAGACAATATAATGCATATTTGAAAGTTCAAATCTATTATTACTGAAGGGTAACACAAGAAATCGCTCGGCCGTGAGTCAACGCAATCGTAAGTAGTGAACTATGGAAGAGCACCAGCCCATTAAAGCTAACCCCTTGTTAC

>R=7134_118_0_0_0_CG_25_bps_438

TATTGATAAATGTGTAGTGATATACTAATTAGCATATATCGTATTGGATGATAAGATTAAAGATTAAAAATTATCCAAACTCTTTTCTATATGAGTAGGATGCTATCATTGCATAGAATTGTATATATGCTTATTGTTTAGTAATGTACCCTAAATGAAATGCTTTTACCAATGTATACTAATAAACAATGCTTTATTAGCATAAGCTAATAAAAGGGCTAATCTTTATAATTGTCAAAGTAATCCTTGAAAGATGTAATAAATATAAATCCTACAGATAGAAAGAATGAGGATATTCCTCCTTTCCTTTAATCCCCAAAATGATATATCTTCAAAATTCTTTTTTTTGTGGTGACATTTTTTTGGAAATAGCTTTATGCGTTAATTTTTGTGATTCGTAATTCCTAAATGATTATGCTATGCTAATATTGTTTAATT

>R=7207_120_0_0_0_CG_44_bps_339

GTCGGAATGTTGTAAAGAAATTATGCGCAAATGTCGCAACATCGTTGAGAACTTCTAGTTTGGTAGTTGAATCAATCTGTCTGGAATGTCAAATCATTCCAACAAAAAAGGAGCTAGGATCAGTCATCCCAGCTCCTTCAGTTATCCTAGCTCGTTTACCAGATCAAAAAGCTCGAGCAATCCGACAGATCGCTCAGTCACTCAATCGGGCAGCTCGGGCAAGCATTCAAAACGACATATTGTCAGACACTCAATTTGACAACGGTTTAACGGCCGAGAAGCGGCAATACGACAGTTCAATAGTACGAGCGACAAGACCGTTTGGTATCCTTATCGTGT

>R=7263_56_0_0_0_CG_30_bps_565

ACTCAATCGTGTAAAAATGTCCTTTCCTTTGATATTGAAATTACCCACATCCGCTAGCTAATTTCCATACAAACTATGAGAAAGCTGTACGTCTAGTAAGATGATTGCCAGCAATTTCCTCTTCATATAAAGAAGAAATCTAGTCTTTTATACAATTTTCTCTTGCACCAGGAAATTATTATCAGCTTTGTGAAGACCTTATATGAAGTTATTCCCTTGTCGTAAGCCCAACCCACTTATGGGCAATATAAAGGCGCCAAGAAGGAGATTCAATGCATTCTTAAAGAAAATCTTGCTTGAAGATCAAATCAGCGCCATCTTCGTCTCGCTCAACTCGACTATTGACTGTTCGTTTAAATTAACTGTACTCCGTATGCCATCTTCTAATTTCATTATCAAATACTAAGGTAATAAAATTTTCTAAAGTAATAAAATTTTTTGAAATTTCGATTCAGGAAAATTTAAATAAAAATTTTTTTAACAAAATAGTAATTGAAAAAAAAATGAAAATATTTCTCTATCAAATGTCTGAATGAAAAAACAAATGAGAAGTTAGCTATTCATT
